# Anti-inflammatory potential of Portuguese thermal waters

**DOI:** 10.1038/s41598-020-79394-9

**Published:** 2020-12-18

**Authors:** A. Silva, A. S. Oliveira, C. V. Vaz, S. Correia, R. Ferreira, L. Breitenfeld, J. Martinez-de-Oliveira, R. Palmeira-de-Oliveira, C. M. F. Pereira, A. Palmeira-de-Oliveira, M. T. Cruz

**Affiliations:** 1 Faculty of Medicine, Center for Neuroscience and Cell Biology, University of Coimbra, Rua Larga, Polo I, 1st Floor, 3004-504 Coimbra, Portugal; 2 Health Sciences Research Centre (CICS-UBI), University of Beira Interior, Av. Infante D. Henrique, 6200-506 Covilhã, Portugal; 3 Faculty of Health Sciences, University of Beira Interior, Av. Infante D. Henrique, 6200-506 Covilhã, Portugal; 4 Labfit–Health Products Research and Development Lda, Ubimedical, Covilhã, Portugal; 5 Centre for Innovative Biomedicine and Biotechnology (CIBB), University of Coimbra, Pólo das Ciências da Saúde, Azinhaga de Santa Comba, 3000-548 Coimbra, Portugal; 6 Faculty of Pharmacy, University of Coimbra, Pólo das Ciências da Saúde, Azinhaga de Santa Comba, 3000-548 Coimbra, Portugal; 7 Faculty of Medicine, Center for Neuroscience and Cell Biology, University of Coimbra, Polo 3, IBILI 3rd Floor, Azinhaga de Santa Comba, 3000-548 Coimbra, Portugal

**Keywords:** Public health, Quality of life, Therapeutics, Skin diseases

## Abstract

In light of Medical Hydrology, thermal waters (TW) are all-natural mineral waters that emerge inside a thermal resort and have therapeutic applications. Their beneficial effect has been empirically recognized for centuries, being indicated for symptom alleviation and/or treatment of several diseases, almost all associated with inflammation. Indeed, an anti-inflammatory effect has been attributed to many different Portuguese TW but there is no scientific validation supporting this empiric knowledge. In the present study, we aimed to investigate the anti-inflammatory properties of 14 TW pertaining to thermal centers located in the Central Region of Portugal, and grouped according to their ionic profile. Mouse macrophage cells stimulated with lipopolysaccharide (LPS), a Toll-like receptor 4 agonist, were exposed to culture medium prepared in TW. Metabolism, nitric oxide (NO) production, inducible nitric oxide synthase (iNOS) expression levels and the scavenging capacity of TW, were investigated in vitro. 11 out of 14 TW reduced NO production and/or iNOS expression, and/or scavenging activity, in macrophages exposed to LPS. The sulphated/calcic TW did not show any effect on at least one of the inflammatory parameters evaluated. Two sulphurous/bicarbonate/sodic TW and the sulphurous/chlorinated/sodic TW promoted an increase in NO production and/or iNOS expression. Our results validate, for the first time, the anti-inflammatory properties of Portuguese TW, supporting their therapeutic use in the treatment of inflammation-related diseases and promoting their putative application in cosmetic products and medical devices.

## Introduction

As stated in the European legislation (2009/54/EC Directive)^1^ and adopted by national organizations^2,3^ “‘Natural mineral water’ means microbiologically wholesome water (…) originating in an underground water table or deposit and emerging from a spring tapped at one or more natural or bore exits”, which could have therapeutic properties or just health beneficial effects^4^.

Natural mineral waters are characterized in terms of their geological, hydrological, physicochemical, microbiological and, eventually, pharmacological and physiological characteristics as well as clinical effects (reviewed by Quattrini et al.^5^). These waters can be classified according to their major physicochemical properties, including pH (acid, pH < 7 or alkaline, pH > 7), temperature at source and mineralization^1^. Regarding water temperature at emergence site and mineralization, there is some controversy about temperature range (revised by Cantista et al.^4^) and mineral concentration values^3–5^. For these reasons, we used the classifications from ‘Instituto de Hidrologia de Lisboa’ (*Hydrology Institute of Lisbon*) for water temperature^6^ and for total mineral content, both adopted by ‘Associação das Termas de Portugal’^3^ (*Portuguese Thermal Center Association*), described in detail in Supplementary data online.

Currently, and in the light of Medical Hydrology, it is usually termed ‘thermal waters’ (TW) all natural mineral waters, independently of their temperature at source, as long as emerging inside a thermal resort and having therapeutic applications^3,7^. The classification based on water mineral content is commonly used to identify their specific therapeutic indications^5^. The associated health benefits to TW use mostly derive from empiric and human trial-based studies and have been extensively reviewed elsewhere^5,8–12^. In Table 1 it is summarized the type of TW, the respective main chemical composition and general therapeutic indications.

**Table 1:** TW chemical types, main chemical content and therapeutic indications. Examples of chemical-associated biological functions are given. Adapted from^4,5,8^.

TW chemical type	Main chemical composition and general therapeutic indications
Sulphurous	**S or H**_**2**_**S, HC0**_**3**_^**−**^**, Na**^**+**^ Considered antiseptic, desensitizer and anti-oxidant waters due to its oxi-reduction capacity Indicated for: reumathic and musculo-skeletic, respiratory, dermatologic and gynaecologic diseases
Carbogaseous	**HC0**_**3**_^**−**^**, Na**^**+**^ Naturally carbonated waters, have high levels of free CO_**2**_. Capable of increasing gastric and intestinal secretions and lowering blood pressure Indicated for: digestive and circulatory systems
Bicarbonated	**HC0**_**3**_^**−**^ Considered anti acidic and alkalizer waters due to its reach composition in alkaline ions (**Ca**^**2+**^, **Mg**^**+**^, **K**^**+**^); capable of alkalize the urine and the blood and to increase the secretions of the pancreas and intestines Indicated for: digestive, respiratory, nephron-urinary and endocrine/metabolic diseases
Chlorinated	**Cl**^**−**^**, Na**^**+**^ Considered anti-inflamatory, antipyretic and disinfectant; capable of stimulating gastric and intestinal secretions and motility; favorable to cicatrization and bone-related afllictions Indicated for: dermatologic, respiratory, digestive, reumathic and musculo-skeletic, and gynaecologic diseases
Sulphated	**SO**_**4**_^**2**−^, **Ca**^**2+**^ Considered hepatoprotective and capable of stimulate intestine peristaltic movements and bile release Indicated for: digestive, nephron-urinary and metabolic/endocrine diseases
Hyposaline	Indicated for: - Nephron-urinary system - Metabolic/endocrine diseases (dependent of predominant ions) - Blood diseases—therapeutic for various types of anemia (Ferric (**Fe**^**2+**^) waters) - Dermatologic and gynaecologic diseases (Silicated waters)
Chemical-associated biological functions	**Bicarbonate (HC0**_**3**_^**−**^**)**, neutralizes gastric acidity, promotes the release of digestive hormones and the reduction of postprandial lipaemia **Calcium (Ca**^**2+**^**)**, essential to bone development, participates in muscle contraction regulation and myocardium activity, blood clotting, nerve impulses transmission and regulation of cell permeability **Chloride (Cl**^**−**^), involved in hydrochloric acid formation important for the digestion process **Iron (Fe**^**2+**^**)**, important for blood and muscle tissues **Magnesium (Mg**^**2+**^**)**, important for bone development, lipids metabolism, protein synthesis, nervous and muscular activities, promoting cardiovascular health **Potassium (K**^**+**^**)**, involved in muscular and neuromuscular activities, acid–base balance, water retention and osmotic pressure **Sodium (Na**^**+**^**)**, important to cell permeability and body fluids. Importantly, excessive ingestion may induce high blood pressure **Sulphur (S), Hydrogen Sulphide (H**_**2**_**S)**, **Sulphate (SO**_**4**_^**2**−^**)** involved in cartilage, hair/nails formation, enzyme activity in redox processes and cellular respiration. **SO**_**4**_^**2**−^ is essential to several metabolic and cellular processes, particularly in foetal growth and development

Portugal has approximately 50 thermal resorts^4^, which display different properties mostly due to their geological variability. This is particularly evident in the northern and center region of Portugal, where the highest diversity in TW properties occurs^4,13,14^. National TW are mainly considered weakly mineralized, sulphurous, bicarbonated or chlorided and sodic^14,15^ and have been indicated as having therapeutic properties for diverse pathologies with particular focus on respiratory and rheumatic/musculoskeletal afflictions. Nevertheless, metabolic-endocrine, gynaecologic, nervous system-related disorders, as well as dermic, gastrointestinal, nephron-urinary and circulatory system diseases are also referred^14,16,17^ (see Table 2).

**Table 2:**
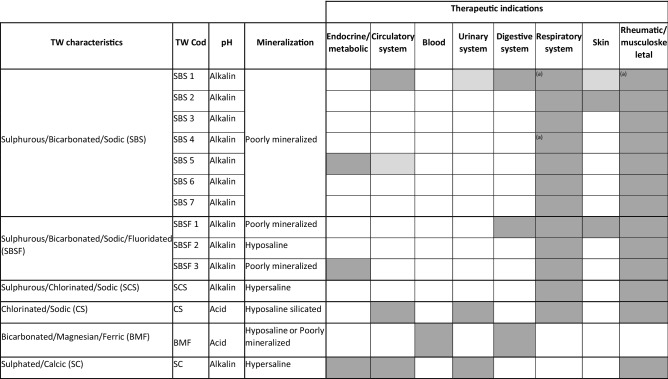
Characterization and therapeutic indications of TW used in this study. Adapted from^4,15–17^.

*Cod* codification: dark grey rectangles—according to Direção-Geral da Saúde (1989)^16^ and Termas de Portugal (2019)^17^; light grey rectangles—according to Termas de Portugal^17^.

^(a)^Not indicated by Termas de Portugal^17^.

Almost all of the above-mentioned pathologies are associated with an inflammatory status. Inflammation is a crucial and complex biological process, carried out by the immune system to maintain normal tissue homeostasis, after injury or infection. Once triggered, it involves immune cell recruitment to the injury site, blood vessels remodeling and the release of molecular proinflammatory mediators. It can be classified as acute (initial and rapid process, with migration of neutrophils and plasma movement towards the damaged site) or chronic (consequence of prolonged inflammation, involving vascular proliferation, mononuclear cells (e.g. macrophages and lymphocytes) and fibroblast recruitment, fibrosis and tissue destruction)^18^. Prolonged inflammation leads to several and etiologically different diseases, such as autoimmune (e.g. multiple sclerosis, rheumatoid arthritis, psoriasis), cardiovascular and brain diseases (e.g. Alzheimer’s and Parkinson’s disease), cancer, diabetes, hepatitis, high blood pressure, atherosclerosis, asthma and allergy conditions, skin conditions (e.g. atopic dermatitis) and reproductive disorders, to name a few^18^. These diseases are associated with an overproduction and release of proinflammatory mediators by macrophages during inflammation, including interleukins (e.g. IL-1, IL-6, IL-8), prostaglandins, tumor necrosis factor alpha (TNF-α), NO and reactive oxygen species (ROS).

Studies addressing the benefits of TW in alleviating the symptoms of inflammation-related diseases have been increasingly reported, mainly in the area of dermatology and rheumatology. Based on human trials, TW use was shown to reduce pain and improve physical function in patients with rheumatic conditions^19–22^. The pain relief is mostly associated with sulphurous TW since sulphur can be absorbed through the skin inducing an analgesic effect (reviewed by Carbajo et al.^23^). In the field of skin disorders, TW-based therapies are mostly recommended for psoriasis and atopic dermatitis conditions^24^. Overall, TW application induced a reduction of scaling, itching and erythema, and an increase in life quality of patients suffering from these conditions^24–29^. Again, sulphurous waters are greatly indicated for the treatment of these and other dermatologic conditions, which seems to be related to their anti-inflammatory, antioxidant, antiseptic and anti-irritation properties^23,24^. Likewise, and based on the latter, these waters are also recommended for the treatment of several respiratory diseases^12,23^. Chronic injury and inflammation lead to airway epithelial cell function dysregulation causing the appearance of lung diseases, such as allergic rhinitis and asthma, characterized by frequent or persistent respiratory limitations. Along with the involvement of inflammatory cells and oxidative stress in the pathogenesis of these diseases, hydrogen sulfide (H_2_S) metabolism is also altered. H_2_S is the most abundant sulfide species present in sulphurous waters and, in healthy conditions, H_2_S-related enzymes (e.g. cystathionine b-synthase and cystathionine g-lyase) are expressed in human lungs, where they exert anti-inflammatory, antioxidant, antibacterial and mucolytic actions, hence contributing to airway epithelium homeostasis^12^. H_2_S-enriched sulphurous water inhalation was shown to have significant clinical efficacy (through the improvement of nasal flow and the reduction of nasal resistance and mucocilliary clearance time) in adult and elderly patients^30^. Salami et al.^31^ showed that inhalation of sulphurous water by children with recurrent upper respiratory tract infections lead to a significant reduction in frequency, duration, severity and social impact of disease episodes. Moreover, the authors suggested that this type of water might have an immunomodulant activity, hence contributing to their therapeutic effects^31^.

TW-based treatments may rely on different factors, such as TW administration techniques and SPA environment, that may account for the improvements observed in human health^32^. With the increased interest of cosmetic industry in commercialized SPA waters as cosmeceuticals, an effort has been made to prove cellular effects, especially in France^33^. Well recognized European thermal centers as *Avène* and *La Roche Posay* have already promoted scientific studies validating their TW effects^27,33–37^. However, in Portugal and to the best of our knowledge, only one study reporting the health benefits of a Portuguese TW has been published^38^. Ferreira et al*.* demonstrated the anti-irritant properties of São Pedro do Sul (SPS) sulphurous TW, in a trial conducted in healthy volunteers exposed to sodium lauryl sulphate. The authors further suggested that SPS TW can help relieve skin irritation and might be included in cosmetic formulations to improve the tolerability of the products^38^.

According to the exposed, biological scientific data supporting the health benefits of Portuguese TW use remain insufficient. Thus, the present study aimed to evaluate the anti-inflammatory properties of these waters in vitro. For that purpose, we analyzed key inflammatory parameters in a mouse macrophage cell line stimulated with LPS, as an inflammation model. NO production and expression levels of iNOS, the enzymve responsible for NO production, and the scavenging capacity of TW in removing NO, were investigated. Our work showed that among the 14 TW used in this study, eight diminished NO production, six decreased iNOS expression, and five exhibited scavenging activity in LPS-exposed macrophages, supporting the anti-inflammatory properties of Portuguese TW.

## Results

### Cell metabolic activity and viability

**Figure 1:**
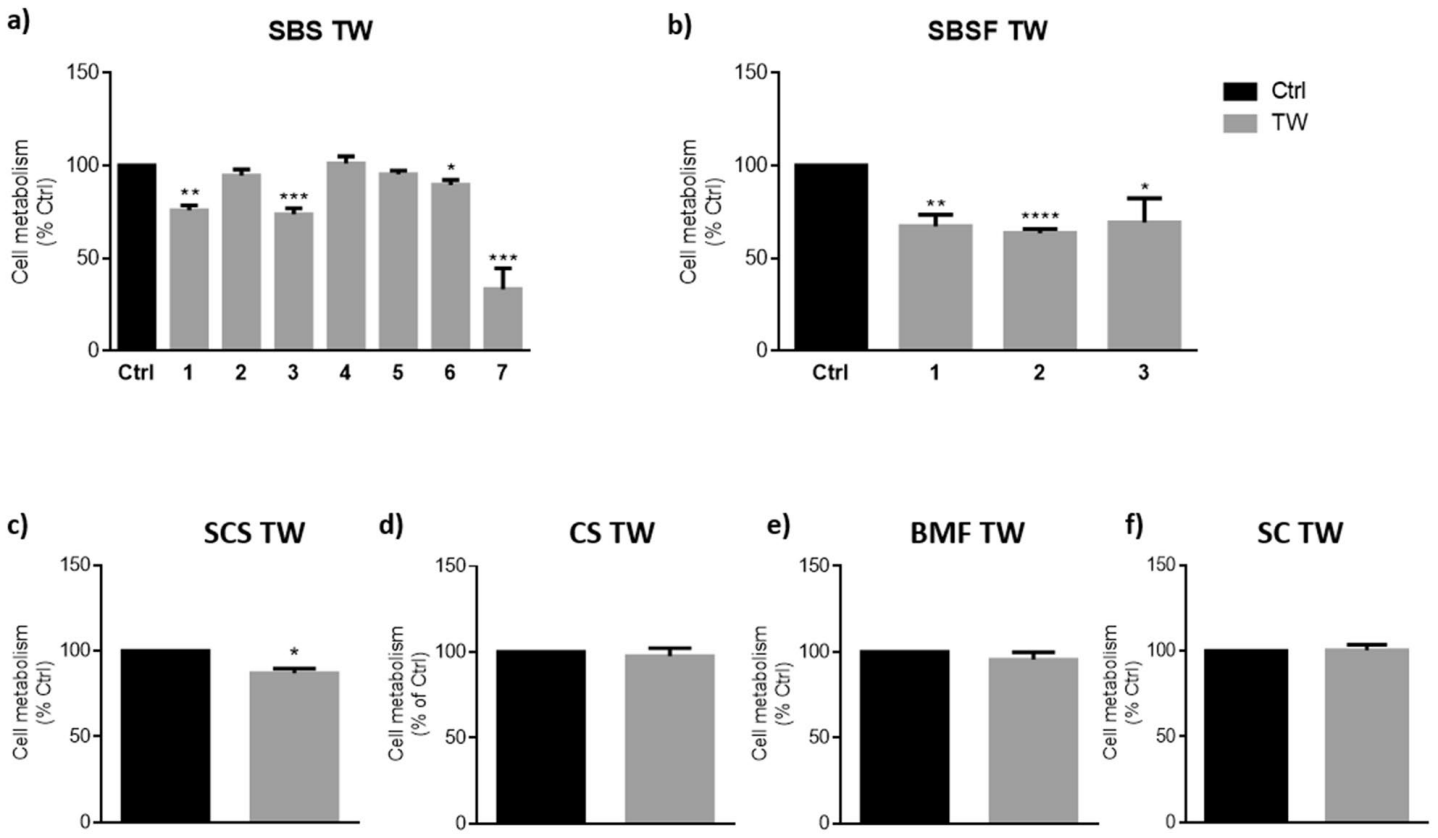
Thermal waters effect on macrophages metabolic state. Cells were plated and exposed to Ctrl medium or SBS (**a**), SBSF (**b**), SCS (**c**), CS (**d**), BMF (**e**) and SC (**f**) thermal waters (TW) for 24 h. Alamar blue assay was further performed to assess cell metabolism. Data correspond to the means ± SEM of at least three independent experiments and are represented as % of control cells (Ctrl, black bars). Statistical analysis: Unpaired t-test; *p* < 0.05 was considered significant: * *p* < 0.05, ** p < 0.01, *** *p* < 0.001, **** *p* < 0.0001, compared to Ctrl. Legend: *SBS* sulphurous/bicarbonate/sodic, *SBSF* sulphurous/bicarbonate/sodic/fluoridated, *CS* chlorinated/sodic, *SCS* sulphurous/chlorinated/sodic, *BMF* bicarbonate/magnesium/ferric, *SC* sulphated/calcic.

In order to determine if the TW affected the metabolic state and/or viability of macrophages after 24 h exposure, we performed the Alamar Blue assay (Fig. 1) and the Trypan Blue exclusion assay (Table 3), respectively. Exposure to TW either did not affect (SBS TW 2, 4 and 5; CS, BMF and SC TW) or significantly reduced cell metabolism around 10–20% when compared to control (Ctrl; SBS TW 1, 3 and 6; SBSF TW 1–3 and SCS TW), with exception of SBS TW 7 that induced more than 50% decrease in metabolic active cells, after 24 h of cell exposure (Fig. 1a). These results seemed to be independent of TW chemical profile, since that among the same group both effects are observed (Fig. 1a), thus corroborating the importance of the individual TW fingerprint.

**Table 3:** Cell viability after 24 h of TW exposure. TW and control values are shown (Ctrl – culture medium, pH 7.2 – 7.4).

	Viability ^(a)^ (Mean ± SEM)	
Sulphurous/Bicarbonated/Sodic	TW	24 h
	Ctrl	83 ± 6
	SBS 1	45 ± 3**
	Ctrl	85 ± 1
	SBS 2	66 ± 4**
	Ctrl	83 ± 6
	SBS 3	54 ± 10 *
	Ctrl	83 ± 6
	SBS 4	37 ± 9**
	Ctrl	66 ± 3
	SBS 5	76 ± 5
	Ctrl	72 ± 5
	SBS 6	78 ± 6
	Ctrl	61 ± 4
	SBS 7	54 ± 9
Sulphurous/Bicarbonated/Sodic/Fluoridated	Ctrl	61 ± 4
	SBSF 1	51 ± 3
	Ctrl	68 ± 4
	SBSF 2	66 ± 3
	Ctrl	61 ± 4
	SBSF 3	45 ± 2*
Sulphurous/Chlorinated/Sodic	Ctrl	58 ± 4
	SCS	36 ± 2**
Chlorinated/Sodic	Ctrl	58 ± 4
	CS	66 ± 9
Bicarbonated/Magnesian/Ferric	Ctrl	67 ± 4
	BMF	63 ± 5
Sulphated/Calcic	Ctrl	58 ± 4
	SC	65 ± 3

Statistics: Unpaired t-test; *p* < 0.05 was considered significant: * *p* < 0.05 and ** *p* < 0.01, compared to Ctrl.

^(a)^Viable cells (% of total cell number) after 24 h of TW exposure.

The decrease in cell metabolism is often accompanied by a decrease in cell viability (Table 3). However, discrepant results regarding cell viability and metabolic activity were obtained for SBS TW 2 and 4 (where metabolism seems to be not affected by TW exposure while viability was significantly reduced); and for SBS TW 6 and SBSF TW 2 (where metabolism is significantly reduced while viability remains unaltered). Nevertheless, the cell metabolism reduction after SBS TW 6 exposure, although statistically significant, reflects only a slight decrease of about ~ 12%, observed both at 24 h and 48 h (Fig. 1 and supplementary Table S2 online), without affecting cell viability and proliferation at both time points (supplementary Table S1 online). These results suggest that SBS TW 6 might slow down cell metabolic activity without compromising cells health state. Concerning SBSF TW 2, the cells displayed a significant metabolism reduction (~ 30%, compared to Ctrl) after 24 h, which did not reflect a decrease in cell viability. Indeed, cell metabolic activity seems to recover with time (~ 20% increase for SBSF TW 2, after 48 h of TW exposure). Interestingly, this was observed for all of TW pertaining to SBSF profile (supplementary Table S2 online), suggesting that cells might need an adaptation period to these TW.

In contrast, cells exposed to SBS TW 2 and 4 exhibited a significant reduction in cell viability and an unaltered metabolic activity, compared to Ctrl (Table 3 and Fig. 1, respectively). However, after 48 h of exposure to SBS2 TW 2, cells were more viable (supplementary Table S1 online), which might indicate that this TW promote an increase in cell metabolism that, in turn, will reflect an increased cell viability overtime. Regarding SBS TW 4, the results are unexpected and difficult to explain, since cell viability and proliferation have a drastic and continued decrease with time (supplementary Table S1 online), while cell metabolism seems to be not affected (Fig. 1a). However, it is important to notice that these results might be due to several factors including the assay itself. In fact, there are some limitations concerning metabolic assays (as Alamar Blue) since they depend on numerous variables, including cell metabolism changes throughout cell lifecycle and chemical dependency on enzymatic metabolic efficiency^39,40^. This might lead to false results, depending on the induction or inhibition of metabolizing enzymes by the compounds tested^39^ (in this case the TW).

### NO production

**Figure 2:**
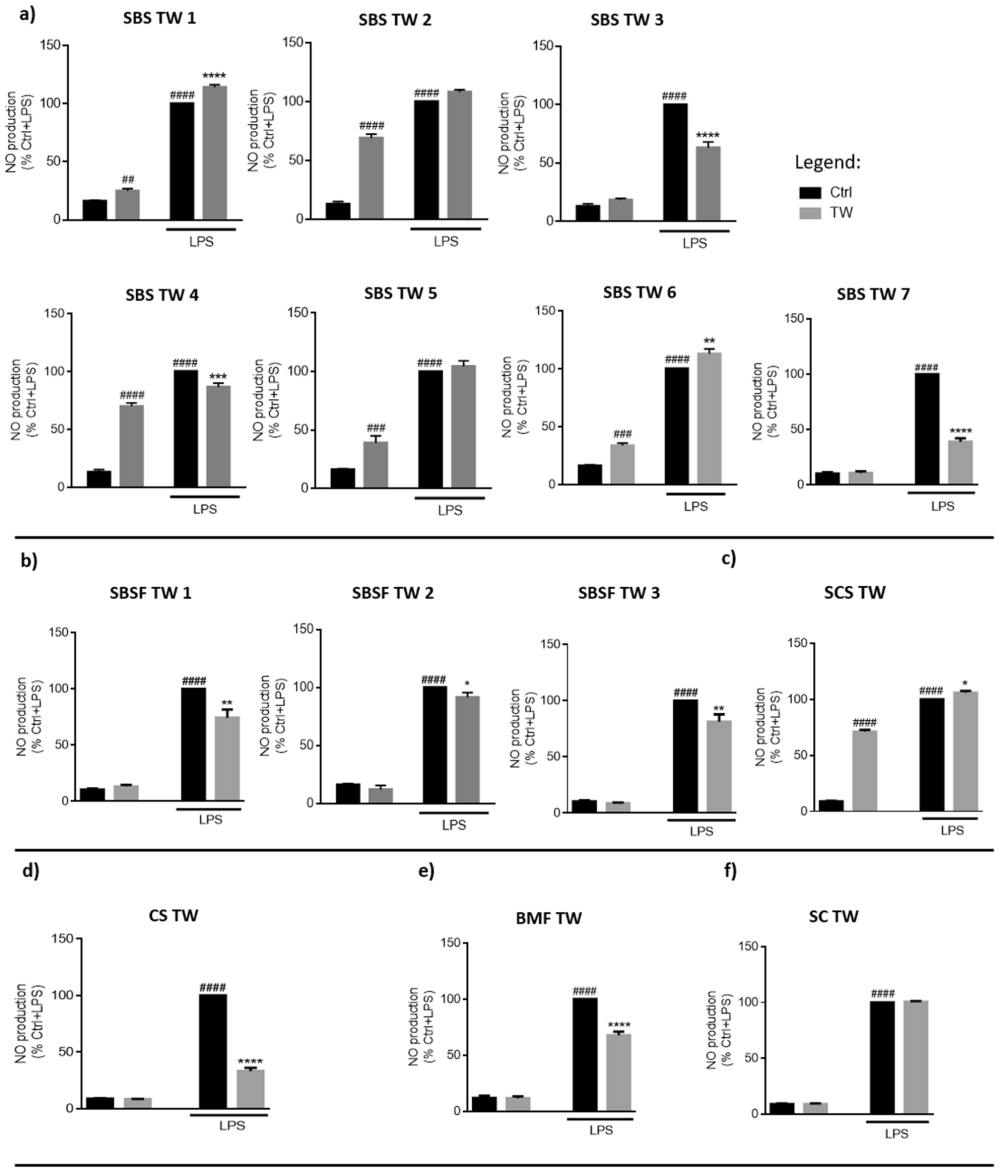
Thermal waters effect on macrophage NO production upon an inflammatory stimulus. Cells were plated and exposed to Ctrl medium or SBS (**a**), SBSF (**b**), SCS (**c**), CS (**d**), BMF (**e**) and SC (**f**) thermal waters (TW) for 24 h, in the presence or absence of LPS (1 µg/mL). Griess assay was performed to assess nitrites levels in the supernatant. Data correspond to the means ± SEM of at least three independent experiments and are represented as % of control (Ctrl) cells exposed to LPS (Ctrl LPS). Statistical analysis: two-way ANOVA with Tukey’s multiple comparison test; *p* < 0.05 was considered significant: * *p* < 0.05, ** p < 0.01, *** *p* < 0.001, **** *p* < 0.0001, compared to Ctrl LPS; ##* p* < 0.01, ### *p* < 0.001, #### *p* < 0.0001, compared to Ctrl. Legend: *SBS* sulphurous/bicarbonate/sodic, *SBSF* sulphurous/bicarbonate/sodic/fluoridated, *CS* chlorinated/sodic, *SCS* sulphurous/chlorinated/sodic, *BMF* bicarbonate/magnesium/ferric, *SC* sulphated/calcic.

Next, we investigated if TW could reduce NO levels produced by macrophages stimulated with LPS (Fig. 2). Interestingly, six TW tested increased NO levels per se, five of which pertaining to the Sulphurous/Bicarbonate/Sodic profile (SBS TW 1–2 and 4–6) and to the Sulphurous/Chlorinated/Sodic (SCS) TW. Nevertheless, some SBS TW (SBS TW 3–4 and 7), all SBSF TW and both CS TW and BMF TW were able to decrease NO levels produced by macrophages in the presence of LPS, suggesting an anti-inflammatory activity upon an inflammatory milieu.

### iNOS expression levels

**Figure 3:**
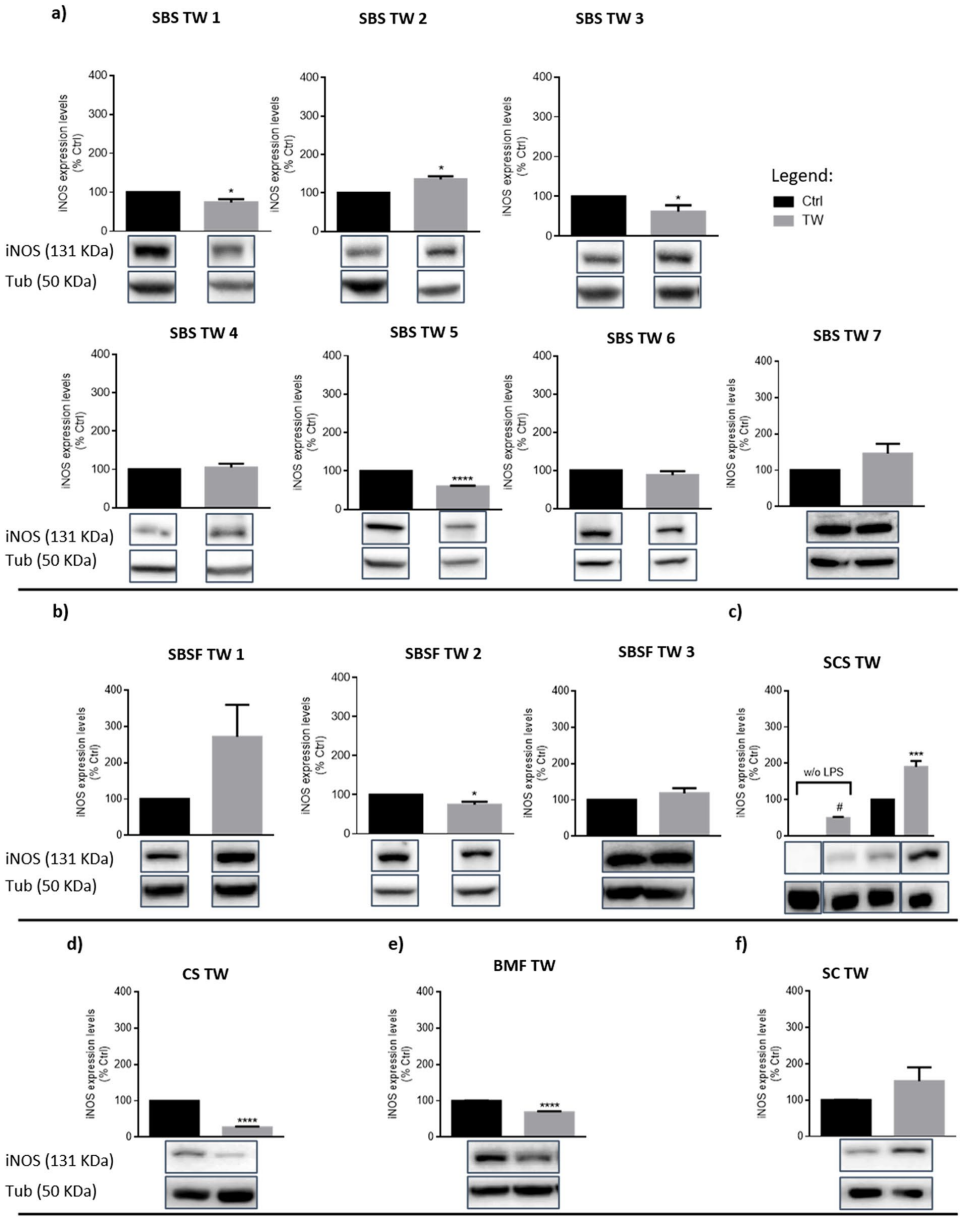
Thermal waters effect on iNOS expression levels of macrophages exposed to an inflammatory stimulus. Cells were plated and exposed to Ctrl medium or SBS (**a**), SBSF (**b**), SCS (**c**), CS (**d**), BMF (**e**) and SC (**f**) thermal waters (TW) for 24 h, in the presence or absence of LPS (1 µg/mL). iNOS and tubulin (Tub) expression levels were determined by Western blotting. iNOS levels were normalized to the loading control Tub. Representative images of western-blots (cropped from different parts of the same/or different gels) are shown. Full-length blots/gels are presented in the supplementary data 2—“Supplementary blots”. Data correspond to the means ± SEM of at least three independent experiments and are represented as % of control cells (Ctrl, black bars) exposed to LPS. Statistical analysis: Unpaired t-test; *p* < 0.05 was considered significant: * *p* < 0.05, *** *p* < 0.001, **** p < 0.0001, compared to Ctrl; #* p* < 0.05, compared to Ctrl without (w/o) LPS. Legend: *SBS* sulphurous/bicarbonate/sodic, *SBSF* sulphurous/bicarbonate/sodic/fluoridated, *CS* chlorinated/sodic, *SCS* sulphurous/chlorinated/sodic, *BMF* bicarbonate/magnesium/ferric, *SC* sulphated/calcic.

The association of changes observed in NO production with alterations in iNOS protein expression levels were then investigated. According to the results, six of the TW evaluated decreased iNOS levels: SBS TW 1, 3 and 5 (Fig. 3a), SBSF TW 2 (Fig. 3b), CS TW (Fig. 3d) and BMF TW (Fig. 2e) while SBS TW 2 and SCS TW upregulated iNOS, in the presence and absence (only SCS TW) of LPS (Fig. 3a,c, respectively). Some of these alterations were correlated with the depletion (SBS TW 3, SBSF TW 2, CS and BMF TW; Fig. 2a,b,d,e, respectively) and rise (SCS TW; Fig. 2c) of NO levels.

### TW scavenging activity

**Figure 4:**
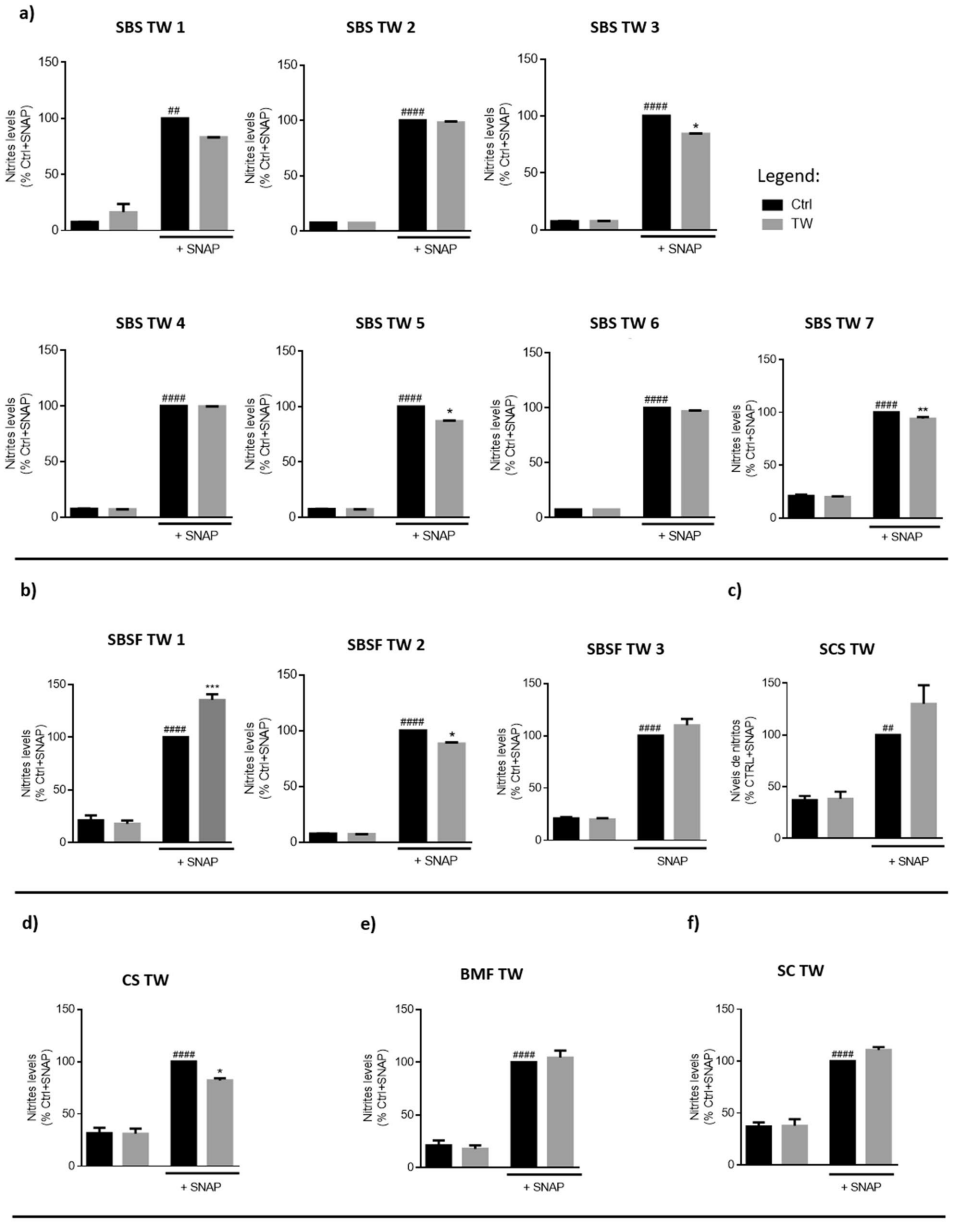
Thermal waters NO scavenging capacity. A NO donor, S-nitroso-N-acetylpenicillamine (SNAP, 300 µM) was added to Ctrl medium or medium prepared with SBS (**a**), SBSF (**b**), SCS (**c**), CS (**d**), BMF (**e**) and SC (**f**) thermal waters (TW), for 3 h. Griess assay was performed to assess nitrites levels in the supernatant. Data correspond to the means ± SEM of at least three independent experiments and are represented as % of Ctrl medium with SNAP (Ctrl). Statistical analysis: two-way ANOVA with Tukey’s multiple comparison test; *p* < 0.05 was considered significant: * *p* < 0.05, ** p < 0.01, *** *p* < 0.001, compared to Ctrl + SNAP; ##* p* < 0.01, #### *p* < 0.0001, compared to Ctrl. Legend: *SBS* sulphurous/bicarbonate/sodic, *SBSF* sulphurous/bicarbonate/sodic/fluoridated, *CS* chlorinated/sodic, *SCS* sulphurous/chlorinated/sodic, *BMF* bicarbonate/magnesium/ferric, *SC* sulphated/calcic.

We also evaluated the scavenging capacity of TW, analyzing their ability to diminish nitrites levels in the medium. As we can observe in Fig. 4, five TW (SBS TW 3, 5 and 7; SBSF TW 2 and CS TW) displayed this capacity and decreased nitrites accumulation in the presence of the NO donor, SNAP. In contrast, one TW, SBSF TW 1 (Fig. 4b), promoted an increase in the medium nitrite levels.

## Discussion

The present study was aimed at conferring scientific validation to the empirically recognized anti-inflammatory effects of Portuguese TW. For the first time, the anti-inflammatory properties of TW from 14 Thermal Centers located in the Central Region of Portugal were validated. Metabolism, NO production, iNOS expression levels and the NO scavenging capacity of TW were investigated in an in vitro model of inflammation, consisting of a mouse macrophage cell line stimulated with the Toll-like receptor 4 agonist, LPS. From the 14 TW studied, 11 promoted a reduction in NO production and/or iNOS expression, and/or exhibited NO scavenging activity in macrophages exposed to LPS, supporting their anti-inflammatory properties.

In Europe, important thermal centers as *Avène* and *La Roche Posay* have already promoted scientific studies validating their TW effects^33–37^. *Avène* TW was shown to protect cell membranes, genomic DNA and proteins of human keratinocytes in a UVA-induced oxidative stress cell model, as mentioned by Merial-Kieny et al.^35^, supporting its antioxidant properties. Anti-allergic effect was also assessed, in which *Avène* TW inhibited histamine and prostaglandin D_2_ release by rat mast cells exposed to substance P- or antigen-induced cells degranulation^36^. The anti-inflammatory effect was also demonstrated in a model of human skin explants stimulated by VIP (vaso-active intestinal peptide), a neurotransmitter that induces vessel dilation. *Avène* TW significantly reduced the area of dilated vessels (compared to distilled water) and decreased TNF-α release^37^. In a comparative study, Zoller et al.^33^ demonstrated that *La Roche Posay* and *Avène* thermal waters both inhibited an inflammatory cytokine (IL-6) and ROS formation in a human keratinocyte cell line irradiated with UVB. However, in Portugal there is a considerable lack of scientific data supporting the health benefits of Portuguese TW; hence, we proposed to evaluate their potential anti-inflammatory properties. We mainly focused in the inflammatory parameters NO and iNOS since this NO producing enzyme is one of key mediators of immune activation and inflammation, and iNOS overexpression has been implicated in numerous human diseases with an inflammatory component^41^. iNOS is one of the three isoforms of the NOS family, namely endothelial NOS (eNOS), neuronal NOS (nNOS) and iNOS. All three enzymes produce NO from L-arginine. iNOS is expressed in many types of cells (e.g. monocytes, mast cells and macrophages) in the presence of inflammatory stimuli, such as bacterial lipopolysaccharides, neuropeptides and immunostimulatory cytokines, producing large quantities of NO through a calcium-independent mechanism^42^. NO is an important signaling molecule, which has numerous molecular targets and plays many different roles in the organism, such as neurotransmission, vascular tone and gene transcription regulation, as well as protein post-translational modifications (reviewed by Förstermann and Sessa^42^). Moreover, NO is important to maintain and regulate skin and its milleu. NO production dysregulation leads to the appearance of several dermatologic diseases and NO has been indicated as a potential antimicrobial agent to be applied in the treatment of skin disorders^43^.

Due to logistic issues, we could not have access to the same number of TW pertaining to the same chemical profile. Hence, seven Sulphurous/Bicarbonate/Sodic TW, three Sulphurous/Bicarbonate/Sodic/Fluoridated TW and one TW of each Chlorinated/Sodic, Sulphurous/Chlorinated/Sodic, Bicarbonate/Magnesium/Ferric and Sulphated/Calcic profile were included. Nevertheless, and as we can conclude from the results summarized in Table 4, a particular TW chemical profile does not reflect the same outcomes regarding the bioactivity, reinforcing the idea that each TW have a unique fingerprint^8^.

**Table 4:** Results summary.

TW	Metabolism	Anti-inflammatory parameters		
		NO production	iNOS expression	Scavenging activity
SBS 1	↓	↑	↓	
SBS 2			↑	
SBS 3	↓	↓	↓	+
SBS 4		↓		
SBS 5			↓	+
SBS 6	↓	↑		
SBS 7	↓	↓		+
SBSF 1	↓	↓		-
SBSF 2	↓	↓	↓	+
SBSF 3	↓	↓		
SCS	↓	↑	↑	
CS		↓	↓	+
BMF		↓	↓	
SC				

(↓) = decrease and (↑) = increase of the evaluated parameters.

+  = positive activity;—= negative activity; the blank rectangles indicate the lack of statistically significant activity.

Three of the TW showed an effect towards a pro-inflammatory state by increasing NO production (SBS TW 6; Fig. 2a) or iNOS expression (SBS TW 2; Fig. 3a) or both (SCS TW; Figs. 2c and 3c), in LPS-treated macrophages. One TW (SC TW) did not induce any alterations on at least one of the inflammation parameters evaluated. Interestingly, SC TW has not been therapeutically indicated for the treatment of respiratory, rheumatic/musculoskeletal and skin disorders (Table 2), all bearing a strong inflammatory component, which is in accordance with our results. Moreover, SC TW was the only one that enhanced macrophage metabolic activity, viability and proliferation, observed both at 24 and 48 h after TW exposure (see supplementary Tables S1 and S2 online), supporting the use of this TW in regenerative skin processes.

In contrast, the sulphurous TW SBS 2 and 6 were expected to reduce some of the inflammation mediators studied since Sulphur-containing waters are described to decrease inflammation^23^. Surprisingly, both TW increased macrophage NO production in the absence of LPS, as well as other four sulphurous TW (Fig. 2a,c). Prandelli et al.^44^ also observed a slight but significant release of pro-inflammatory mediators (namely the neutrophil attracting chemokine CXCL8, TNF-α and IL-6) in human monocytes exposed to a sulphurous TW. The authors suggest that depending on ion and oligoelement composition of TW, some S-based compounds may induce a mild pro-inflammatory effect, which would alert the immune system to immediately respond against infections^44^. This mechanism would validate the efficacy of TW also in preventing infectious-related disesases. In fact, our team have already investigated the antimicrobial effect of these TW, and sulphurous TW have proved to exhibit anti-microbial properties against a range of different bacteria^45^. Accordingly, it was demonstrated that H_2_S augments NO levels in keratinocytes, through iNOS increase and in a protein kinase B (Akt) gene- dependent way^46^, which might contribute to the sulphurous TW antimicrobial activity previously reported^23^. However, an increase in iNOS expression in macrophages in the absence of LPS was only observed for SCS TW (Fig. 3c), thus explaining the rise of NO levels (Fig. 2c). Furthermore, SCS TW was unable to scavenge NO radical (Fig. 4c) and alterations in NO and iNOS were exacerbated in the presence of LPS, suggesting that SCS TW does not exhibit an anti-inflammatory effect but rather exhibits an anti-microbial activity (e.g. against *Cutibacterium acnes*, as shown by us)^45^.

In contrast, the production of NO by macrophages observed when the cells were exposed to SBS TW alone was not always accompanied with an increase in iNOS expression (Fig. 2 and Fig. 3). However, Chang et al.^47^, demonstrated a novel negative feedback mechanism by which NO downregulates iNOS gene expression by preventing NF-kB activation^47^. This also could explain the down-regulation of iNOS in cells exposed to SBS TW 1, in the presence of LPS.

Interestingly, cells exposed to SBS 4 TW were able to reduce LPS-induced NO production by macrophages, despite the increased levels of NO observed in the absence of LPS (Fig. 2a). NO depletion was also observed in cells stimulated with LPS and exposed to SBS TW 7 and BMF TW (Fig. 2a,e, respectively) that was consistent with SBS TW 7 capacity in removing NO (Fig. 4a), and with iNOS protein levels reduction promoted by BMF TW (Fig. 3e). Robust results were obtained for SBS TW 3, SBSF TW 2 and CS TW, regarding that all three reduced NO (Fig. 2a,b,d, respectively) and iNOS (Fig. 3a,b,d, respectively) expression levels in the cells exposed to LPS, and displayed scavenging capacity (Fig. 4a,b,d, respectively). Altogether, the results suggest that these TW hold strong anti-inflammatory properties and should be indicated for the treatment of inflammatory diseases.

It is important to emphasize that the present study focused on relevant but not all inflammation-related mediators and molecular events, hence not disclosing the existence of others being affected by TW exposure. The results herein presented intended to scientifically justify the anti-inflammatory effect attributable to Portuguese TW without scrutinize the associated mechanisms, which was beyond the scope of this study. However, a deeper investigation about the molecular and cellular pathways associated to TW anti-inflammatory properties, using other cell lines and more physiologic cellular models (e.g. 3D models), based on a methodology specifically designed to the study of a particular disease, should be performed.

In addition, examining the existence of a particular TW microbiome and its byproducts, which might influence cellular behavior, would be of utmost importance. Martin et al. (2016) observed that an aqueous protein extract from Aquaphilus dolomiae (a bacterium isolated from Avène Spring water) induced the secretion of IL-10 (an anti-inflammatory cytokine) in human monocyte-derived dendritic cells^48^. Thus, besides the existence of TW ions that have an important role in preventing inflammation, such as zinc^49^ and magnesium^50^, one cannot exclude other factors contributing to TW effects.

Overall, our results greatly contributed to the scientific validation of the anti-inflammatory properties of Portuguese TW, particularly of SBS 3, SBSF 2 and CS TW, supporting their therapeutic use for the treatment of inflammatory-related diseases and further application in cosmetic products and medical devices.

## Materials and methods

### Materials

Water containers were purchased from VWR (Alfragide, Portugal). Raw 264.7 cell line was acquired from American Type Culture Collection (ATCC-TIB-71) and kindly supplied by Dr. Otília Vieira (Center for Neuroscience and Cell Biology, University of Coimbra, Coimbra, Portugal). Dulbecco's Modified Eagle Medium (DMEM) and fetal bovine serum (FBS) were purchased from Thermo Fisher Scientific (Massachusetts, USA). LPS from *Escherichia coli* (serotype 026:B6) was obtained from Sigma-Aldrich Corp. (St. Louis, MO, USA). The protease and phosphatase inhibitor cocktails were obtained from Roche (Mannheim, Germany). Acrylamide was purchased from Promega (Madison, WI, USA) and the Polyvinylidene difluoride (PVDF) membranes were from Millipore Corp. (Bedford, MA, USA). The primary antibody mouse anti-iNOS was from R&D Systems (Minneapolis, MN, USA) and mouse anti-β-Tubulin was obtained from Sigma-Aldrich Corp. (St. Louis, MO, USA). The anti-mouse Horseradish Peroxidase conjugated secondary antibody was purchased from Santa Cruz Biotechnology (Heidelberg, Germany). Enhanced chemiluminescence (ECL) substrate was obtained from Bio-Rad (Hercules, USA). All other reagents were purchased from Sigma-Aldrich Corp. (St. Louis, MO, USA).

### Methods

#### Thermal centers recruitment and water collection

14 Thermal Centers belonging to the Central Region of Portugal were enrolled in this study. TW were collected from the spring/borehole of each Thermal Centre, after purging and according to the appropriate procedures^51^. Briefly, each TW was collected in several sterile flasks that were immediately sealed with parafilm and transported refrigerated in thermal boxes with frozen accumulators. The flasks were further maintained in the lab at 4 °C, in the dark, and each flask was opened just once to avoid TW composition alterations. Upon TW receival, one of the flasks was used to measure TW pH, osmolality and organoleptic features (i.e., odor, color, deposit and aspect). All other physic-chemical parameters were periodically measured at the thermal center who gave us the more recent certified analytical reports (see supplementary Table S3 online). To ensure the confidentiality of the Thermal Centers involved, each flask was uniquely labelled. TW were grouped according to their ionic profile and as stated by ‘Termas de Portugal’^17^, in six groups. A summary of the characteristics and therapeutic indications of the TW used in this study are shown in Table 2.

#### Cell culture and TW exposure

Raw 264.7, a mouse leukaemic monocyte macrophage cell line, was cultured in DMEM (powder, low glucose with pyruvate and L-Glutamine), supplemented with 10% (v/v) of non-inactivated FBS, 100 U/ml penicillin and 100 μg/ml streptomycin, D-Glucose (up to the final concentration of 4.5 g/L) and sodium bicarbonate (1.5 g/L), at 37 °C in a humidified atmosphere of 95% air and 5% CO2. Every two days, before reaching confluence, the cells were passaged using a cell scrapper to detach the cells, which were further subcultured in fresh culture media according to ATCC recommendations.

To determine the effects of each TW on cells, culture medium was prepared using TW instead of ultrapure water (control). After the culture medium components were dissolved, either in TW or ultrapure water, the medium was filtered with a sterile 0.2 μm membrane. TW-containing media was prepared from a newly opened flask each time needed.

#### Alamar Blue assay

Cell metabolism was assessed using Alamar Blue (resazurin) reduction colorimetric assay, as previously described^52^. Briefly, cell duplicates were plated in a density of 2.50 × 10^5^ cells/mL, in a 96-well plate with a final volume of 0.2 mL/well, and exposed to TW, for 24 h. 50 µM of resazurin solution (in sterile PBS) was added to each well for 4 h, after 20 h of TW exposure. Absorbance was read at 570 and 620 nm with a Synergy HT multi‐mode microplate reader (BioTek, Bad Friedrichshall, Germany). Metabolically active cells reduce resazurin (a non-fluorescent blue dye) into resorufin (pink colored and fluorescent form) and, hence, their number correlates with the magnitude of dye reduction. Results were expressed as a percentage of control.

#### Cellular proliferation and viability (Trypan Blue dye exclusion assay)

Cells were plated at a density of 2.50 × 10^5^ cells/mL, in a 6-well plate in a final volume of 2 mL/well, and exposed to TW (and pH control, when applicable), for 24 h. Cells were detached using a scrapper and 0.01 mL of 0.4% (w/v) trypan blue stock solution in PBS was added (1:1 ratio). The number of viable cells was counted manually using a Neubauer chamber.

#### Determination of nitric oxide (NO) production

Cells were seeded at a density of 2.50 × 105 cells/mL in 48-well plates with a final volume of 0.6 mL/well and allowed to stabilize overnight (O/N). Then, the cells were simultaneously exposed to LPS (1 µg/mL) and TW, for 24 h. NO production was further determined by measuring nitrite accumulation in the culture supernatants, through a colorimetric assay using the Griess reagent^53^. Briefly, equal volumes of cell culture supernatants and Griess reagent [1% (w/v) sulphanilamide in 5% (w/v) phosphoric acid and 0.1% (w/v) N-(1-naphthyl)-ethylenediamine dihydrochloride] were mixed and incubated at room temperature (RT) for 30 min. The absorbance was measured at 550 nm in a Synergy HT multi‐mode microplate reader (BioTek, Bad Friedrichshall, Germany). Nitrite concentration was calculated through a regression analysis of a sodium nitrite standard curve.

#### Cell lysates and western blotting

Cells plated in a density of 3.50 × 10^5^/mL were simultaneously exposed to LPS (1 µg/mL) and TW, for 24 h. Total cell lysates were obtained as previously described by us^54^, with modifications. Cells were lysed with RIPA buffer (150 mM NaCl; 50 mM Tris–HCl, pH 8.0; 1% Nonidet P-40; 0.5% v/v sodium deoxycholate; 0.1% v/v SDS; 2 mM EDTA), freshly supplemented with DTT (1 mM) and protease and phosphatase inhibitor cocktails. After 30 min on ice, the cells were centrifuged (12,000 g for 10 min, at 4 °C) and protein concentration was calculated using the bicinchoninic acid method. Lysates samples were denatured at 95 °C, for 5 min (in sample buffer: 0.125 mM Tris, pH 6.8; 2% SDS (w/v); 100 mM DTT; 10% glycerol (v/v) and bromophenol blue). 30 µg of protein were separated on a 10% (v/v) sodium dodecyl sulfate (SDS) polyacrylamide gel and transferred to a PVDF membrane. Membranes were further blocked with 5% (w/v) fat-free dry milk in TBS-T (Tris-buffered saline/0.1% Tween 20 (v/v)), for 1 h, at RT and incubated O/N at 4 °C with the primary antibody anti-iNOS (1:1000). After the washing step with TBS-T, the membranes were incubated with anti-mouse horseradish peroxidase conjugated secondary antibody (1:40,000), for 1 h at RT. Membranes were also incubated with a mouse anti-β-tubulin antibody (1:20,000) as a loading control. Blots were visualized by chemiluminescence using ImageQuant LAS 500 (GE Healthcare, Chicago, Illinois, USA) and analyzed using TotalLab TL120 software.

#### Determination of NO scavenging activity

NO scavenging activity was evaluated by adding the NO donor S-nitroso-N-acetylpenicillamine (SNAP, 300 µM) to 300 µL of each culture medium, for 3 h. Nitrite levels in the medium were then quantified by Griess method, as described above.

#### Statistical analysis

The results are presented as mean ± SEM of the indicated number of experiments and were analyzed with t-test or two-way ANOVA with Tukey’s multiple comparision test, using GraphPad Prism (GraphPad Software, La Jolla California USA; www.graphpad.com). *p* < 0.05 was considered significant.

## Supplementary Information

Supplementary Information.
