# Supplementary material for: Anti-inflammatory potential of Portuguese thermal waters
Source: Sci Rep. 2020 Dec 18;10:22313. doi: 10.1038/s41598-020-79394-9 (PMC7749128; doi:10.1038/s41598-020-79394-9)

Anti-inflammatory potential of Portuguese Thermal Waters

***Submitted to* Scientific Reports**

A Silva[1*], AS Oliveira[2], CV Vaz[2], S Correia[2], R Ferreira[2,3], L Breitenfeld[2,3], J Martinez-de-Oliveira[2,3], R Palmeira-de-Oliveira[2,3,4], C MF Pereira[1,5], A Palmeira-de-Oliveira[2,3,4], MT Cruz[1,6*].

*Corresponding authors

Ana C. Silva
e-mail: anacrs@cnc.uc.pt

Maria Teresa Cruz
e-mail: trosete@ff.uc.pt

Supplementary information -

Thermal Water classification

Regarding some controversy about temperature range (revised by Cantista 2008-2010) and mineral concentration values (Cantista 2008-2010; Associação das Termas de Portugal 2009; Quattrini et al. 2016), we used the classifications from 'Instituto de Hidrologia de Lisboa' (*Hydrology Institute of Lisbon*) for water temperature (Herculano de Carvalho et al. 1961) and for total mineral content, both adopted by 'Associação das Termas de Portugal' (*Portuguese Thermal Center Association*) (Associação das Termas de Portugal 2009). Hence, natural mineral waters were classified, regarding their emergence temperature (ET), as follows (Herculano de Carvalho et al 1961; Associação das Termas de Portugal 2009):

1) Hypothermal (ET < 25°);
2) Mesothermal (ET between 25-35°C);
3) Thermal (ET between 35-40°C);
4) Hyperthermal (ET > 40°C).

Concerning their total mineralization, natural mineral waters were classified according to Associação das Termas de Portugal (2009) and Cantista (2008-2010), as:

1) Hyposaline waters: total mineralization less than 200 mg/L;
2) Poorly mineralized water: total mineralization between 200 and 1000 mg/L;
3) Mesosaline waters: total mineralization between 1000 and 2000 mg/L;
4) Hypersaline waters: total mineralization above 2000 mg/L.

Supported by the latter classification, Curto-Simões (1993) proposed six classes of Portuguese natural mineral waters, based on its chemical composition (a brief description is given; for a detailed one see refs. Cantista 2008-2010; Associação das Termas de Portugal 2009):

1) Hyposaline waters, which have a total mineralization value < 200 mg/L, divided in non-silicated and silicated;

2) Sulphurous waters, which contain Sulphur (mainly reduced forms) and $HC0_3^-$ and Na^+ as dominant ions;

3) Carbogaseous waters, with > 500 mg/L of free CO_2, have $HC0_3^-$ and Na^+ as dominant ions;

4) Bicarbonated waters that contain $HC0_3^-$ as the dominant ion;

5) Chlorinated (or chloride) waters, which have as dominant ions Cl^- and $Na^{+;}$

6) Sulphated waters, with SO_4^{2-} and Ca^{2+} as dominant ions.

References

- Associação das Termas de Portugal. Manual de Boas Práticas dos Estabelecimentos Termais https://www.dgs.pt/documentos-e-publicacoes/manual-de-boas-praticas-dos-estabelecimentos-termais.aspx (2009).

- Cantista, P. O termalismo em Portugal. An. Hidrol. Medica. 3, 79–107 (2008-2010).

- Curto Simões, M. M. Águas Minerais Portuguesas: da sua caracterização físico-química ao esboço de uma classificação. Boletim de Minas. **30**, 13-26 (1993).

- Herculano de Carvalho, A., Almeida, J. D. & Reis, E. M. Guia de análise química das águas (potáveis, minerais e para a indústria) 162 pp (Associação dos Estudantes do IST, Lisboa, 1961).

- Quattrini, S., Pampaloni, B. & Brandi, M. L. Natural mineral waters: Chemical characteristics and health effects. Clin. Cases. Miner. Bone. Metab. 13, 173–180 (2016).

Supplementary Tables

Table S1 – Cell viability and proliferation after 24 and 48 h of TW exposure. TW and control values are shown (Ctrl – culture medium, pH 7.2 – 7.4).

		Viability [(a)] (Mean ± SEM)		Proliferation [(b)] (Mean (x10^3) ± SEM)	
	**TW**	**24 h**	**48 h**	**24 h**	**48 h**
**Sulphurous/Bicarbonated/Sodic**	Ctrl	83 ± 6	89 ± 2.7	1600 ± 289	2391 ± 175
	SBS 1	45 ± 3	37 ± 5	710 ± 34	502 ± 177
	Ctrl	85 ± 1	84 ± 4	1463 ± 100	3227 ± 354
	SBS 2	66 ± 4	73 ± 8	736± 85	623 ± 50
	Ctrl	83 ± 6	89 ± 2.7	1600 ± 289	2391 ± 175
	SBS 3	54 ± 10	43 ± 1	501 ± 79	556 ± 53
	Ctrl	83 ± 6	89 ± 2.7	1600 ± 289	2391 ± 175
	SBS 4	37 ± 9	13 ± 1	265 ± 47	140 ± 17
	Ctrl	66 ± 3	76 ± 5	727 ± 125	1736 ± 290
	SBS 5	76 ± 5	68 ± 6	391 ± 74	949 ± 257
	Ctrl	72 ± 5	76 ± 3	972 ± 170	2235 ± 432
	SBS 6	78 ± 6	74 ± 5	582 ± 134	859 ± 245
	Ctrl	61 ± 4	73 ± 4	1657 ± 246	4366 ± 428
	SBS 7	54 ± 9	51 ± 9	1133 ± 274	1465 ± 326
**Sulphurous/Bicarbonated/Sodic/Fluoridated**	Ctrl	61 ± 4	73 ± 4	1657 ± 246	4366 ± 428
	SBSF 1	51 ± 3	62 ± 3	1247 ± 339	3100 ± 720
	Ctrl	68 ± 4	76 ± 4	998 ± 190	2448 ± 164
	SBSF 2	66 ± 3	75 ± 8	775 ± 12	1651 ± 148
	Ctrl	61 ± 4	73 ± 4	1657 ± 246	4366 ± 428
	SBSF 3	45 ± 2	58 ± 8	1220 ± 132	2515 ± 104
**Sulphurous/Chlorinated/Sodic**	Ctrl	58 ± 4	77 ± 2	1473 ± 128	5606 ± 864
	SCS	36 ± 2	33 ± 3	1113 ± 121	1593 ± 218
**Chlorinated/Sodic**	Ctrl	58 ± 4	77 ± 2	1473 ± 128	5606 ± 864
	CS	66 ± 9	59 ± 5	983 ± 114	2940 ± 110
**Bicarbonated/Magnesian/Ferric**	Ctrl	67 ± 4	74 ± 6	1506 ± 267	4560 ± 674
	BMF	63 ± 5	72 ± 4	1633 ± 298	3797 ± 555
**Sulphated/Calcic**	Ctrl	58 ± 4	77 ± 2	1473 ± 128	5606 ± 864
	SC	65 ± 3	80 ± 3	1733 ± 107	6993 ± 18

[(a)] Viable cells (% of total cell number); [(b)] Total cell number (live + dead) at 24 and 48 h derived from 500,000 plated cells.

Table S2 – Percentage (%) of cell metabolism after 24 and 48 h of TW exposure compared to control (100%).

	TW	24 h	48 h
**Sulphurous/Bicarbonated/Sodic**	SBS 1	75.87 ± 2.6	82.08 ± 2.4
	SBS 2	94.51 ± 3.4	104 ± 8
	SBS 3	73.69 ± 3.3	73.24 ± 5.2
	SBS 4	101.1 ± 3.7	99.25 ± 5.3
	SBS 5	95.26 ± 1.9	111.8 ± 11.15
	SBS 6	89.6 ± 2.6	88.9 ± 6.9
	SBS 7	24.5 ± 11.8	8.6 ± 5.6
**Sulphurous/Bicarbonated/Sodic/Fluoridated**	SBSF 1	67.3 ± 6.2	90.6 ± 0.3
	SBSF 2	63.62 ± 2.2	83.43 ± 5.3
	SBSF 3	69.3 ± 12.9	81 ± 7.7
**Sulphurous/Chlorinated/Sodic**	SCS	87 ± 2.6	54 ± 11
**Chlorinated/Sodic**	CS	97.6 ± 4.6	113.6 ± 12.2
**Bicarbonated/Magnesian/Ferric**	BMF	95.5 ± 4.3	101 ± 3
**Sulphated/Calcic**	SC	100.3 ± 3.2	88.6 ± 11

Table S3 – TW organoleptic features and detailed physicochemical composition according to the certified analytical reports obtained from the participating thermal centers (adapted from Oliveira et al, 2019[45]).

TW	SBS 1	SBS 2	SBS 3	SBS 4	SBS 5	SBS 6	SBS 7	SBSF 1	SBSF 2	SBSF 3	SCS	CS	BMF	SC
**Organoleptic features**														
Smell*	slight sulphuric	sulphuric	sulphuric	sulphuric	sulphuric	sulphuric	slight sulphuric	sulphuric	slight sulphuric	sulphuric	sulphuric	odorless	odorless	odorless
Deposit*	null	null	null	null	null	null	null	null	null	null	null	null	orange "drops"	null
Aspect*	clear	clear	clear	clear	clear	clear	clear	clear	clear	clear	clear	clear	clear	clear
Color*	colorless	colorless	colorless	colorless	colorless	colorless	colorless	colorless	colorless	colorless	colorless	colorless	colorless	colorless
**Physicochemical constants and non-dissociated substances**														
Emergence Temperature (°C)	NS	NS	46.6	NS	NS	46.5	34.6	47.1	NS	NS	34.8	27	NS	19.3
pH	8.43	7.91	8.60	8.47	7.9	8	8.3	8.9	9.48	8.6	6.93	5.20	6.33	7.2
pH*	8.74	8.12	8.60	8.39	8.3	7.7	8.3	8.92	9.40	9.0	7.27	5.40	6.35	7.4
Osmolality (mosmol/kg)*	17	26	25	28	7	12	14	18	4	13	69	5	13	21
Conductivity (µS/cm)	303	421	544	538	404	506	466	383	206	367	3948	48.1	217	2200
Total Sulphurisation (mL I2 0.01 N/L)	13	16	43	12.4	4.0	20	6	24	7.9	25	57.06	NS	NS	NS
Total Sulphur (mmol/L)	NS	0.22	0.33	0.1	0.06	0.13	0.19	NS	0.18	0.30	NS	NS	NS	NS
Hydrogen sulfide (mg H_2S/L)	<0.5	<0.5	<0.5	2	0.17	<0.5	<0.5	<0.5	<0.5	<0.04	NS	<0.04	NS	NS
Total alkalinity (mg $CaCO_3$/L)	75.5	125	151	186	130	151	127	114	60.5	120	NS	3.5	72.1	210
Total hardness (mg CaCO3/L)	10	12	6.1	12	10.4	13	14	6.5	7.7	7.3	NS	7	80	1700
Silica (mg SiO_2/L)	55	43	60	33	55	87	52	63	33	81	18	10	8	9.8
Dry residue at 180 ºC (mg/L)	226	295	383	87	282	379	322	297	162	292	NS	35	118	2200
Total mineralization (mg/L)	268	369	449	485	373	467	407	343	191	385	2989.6	36	169	2130.8
**Anions (mg/L)**														
Bicarbonate (HCO3-)	82.9	148	151	210	160	176	152	101	51.3	140	314.6	4.3	87.8	250
Carbonate (CO3)	<2	<2	7	3	<1.0	<2	<2	9.8	2.5	2.7	NS	<1	<2	<3
Chloride (Cl-)	26	32	45	51	30	54	51	27	6.9	26	1006.8	9.9	19	27
Fluoride (F-)	16	15	24	11	16	16	17	21	9.8	18	1.46	<0.1	0.2	0.79
Bisulfide (HS-)	2.2	2.7	7.1	2	<0.1	3.3	1.0	4.0	1.3	2.9	4.1	NS	NS	NS
Nitrates (NO^{3-})	<0.3	<0.3	<0.3	<0.1	1.6	<0.3	<0.3	<0.3	<0.3	<0.27	0.31	1.6	<0.3	0.15
Nitrites (NO^{2-})	<0.01	<0.01	<0.01	<0.01	0.05	<0.01	<0.01	<0.01	<0.01	<0.01	NS	<0.01	<0.01	<0.01
Silicate ($H_3SiO_4^-$)	3.4	<1	10	33	1.3	2.7	2.4	16	23	9.1	NS	<1	<1	<2
Sulfate (SO_4^{2-})	7.9	21	12	5.5	1.9	5.3	15	4.1	13	10	NS	1.2	9.6	1200
**Cations (mg/L)**														
Ammonium (NH_4^+)	0.08	0.05	0.7	0.19	0.072	0.43	0.12	0.36	0.11	0.36	0.35	<0.05	<0.05	0.05
Calcium (Ca^{2+})	3.9	4.1	2.5	4.1	4.1	4.8	5.3	2.6	3.1	2.9	270.2	0.66	4.2	560
Lithium (Li^+)	0.3	0.65	0.72	0.31	0.875	1.5	1.0	0.33	0.16	0.719	0.04	<0.1	<0.1	0.026
Magnesium (Mg^{2+})	0.15	0.35	<0.1	0.35	0.047	0.18	0.11	<0.1	<0.1	0.013	57.0	1.3	17	60
Potassium (K^+)	2.0	2.8	4.4	1.8	2.3	4.4	2.4	2.8	0.9	3.3	5.0	0.37	1.4	2
Sodium (Na^+)	67	99	125	130	97	111	108	91	46	88	660.1	6.3	13	21
Iron (Fe^{2+})	<0.01	<0.01	<0.01	<0.025	<0.01	<0.01	<0.01	<0.01	<0.01	<0.002	NS	<0.01	9	<0.025
**Metals (µg/L)**														
Crómium (Cr)	<1,0	NS	< 1,0	NS	NS	<1,0	< 1,0	< 1,0	NS	NS	NS	<1,0	NS	<1,0
Cupper (Cu)	<2	NS	< 2	NS	NS	<2	< 2	< 2	NS	1	NS	21	NS	2,4
Selenium (Se)	<0,4	NS	< 0,4	NS	NS	<0,4	< 0,4	< 0,4	NS	NS	<0,4	<0,4	NS	< 2,5
Zinc (Zn)	<50	NS	< 50	NS	NS	<50	< 50	< 50	NS	<50	60	<50	NS	NS

NS - Data not stated in the certified analytical sheet. *physicochemical parameters measured in our laboratory.

Supplementary data - Uncropped Blots (Fig. 3 – iNOS (131-135 kDa) and Tub (50 kDa) expression levels)

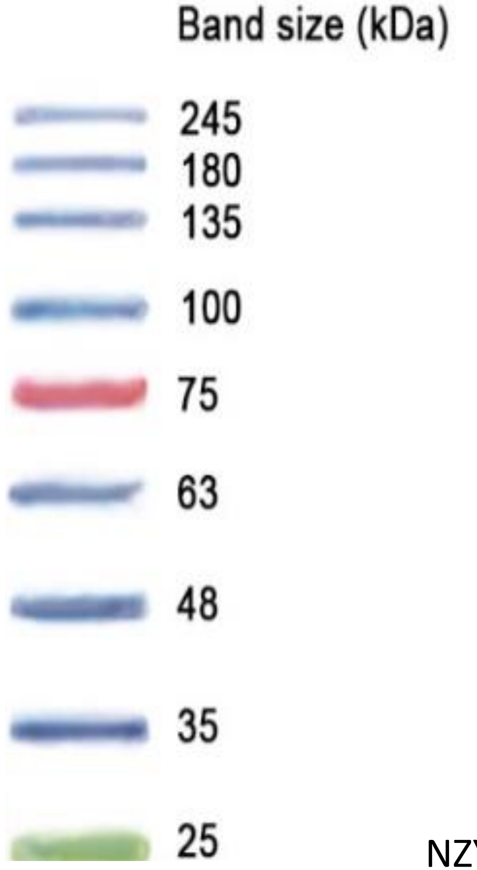

Tubulin loading control was probed for on the same blot of iNOS

NZYColour Protein Marker II

SBS TW 1

INOS

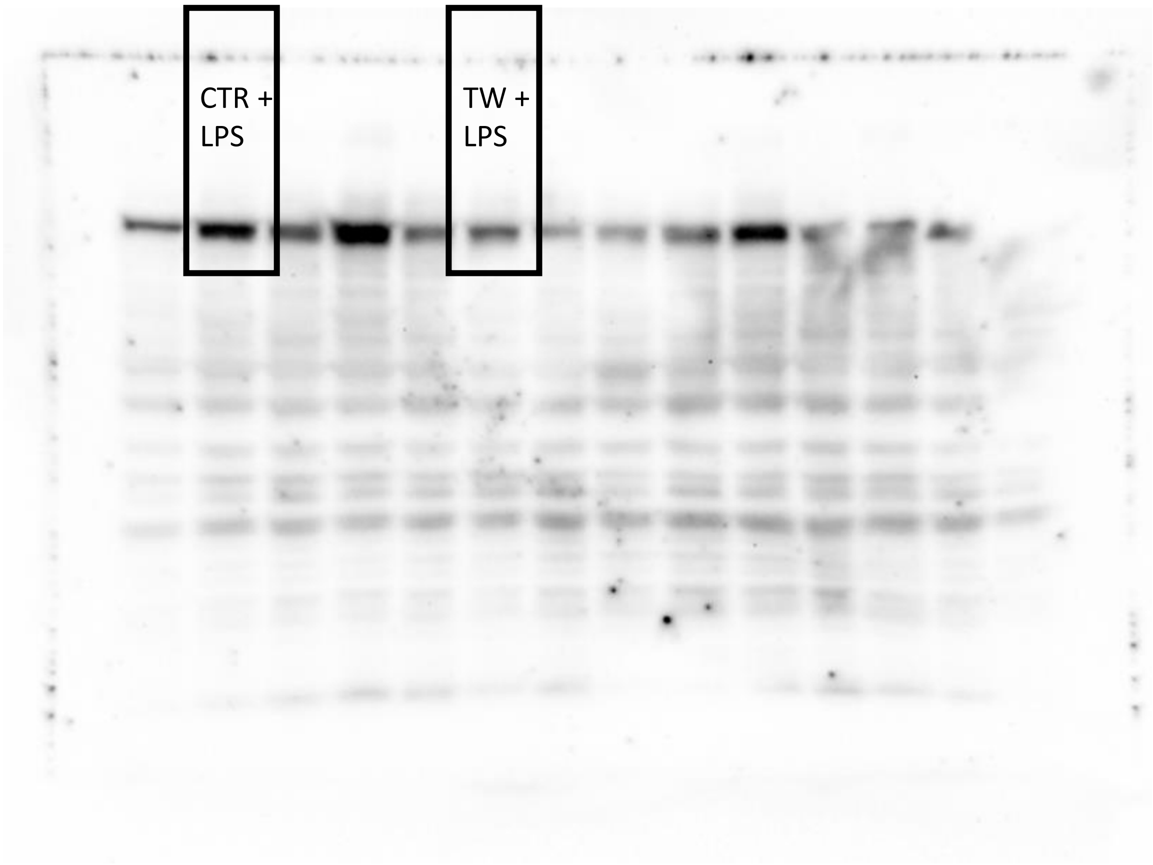

TUB

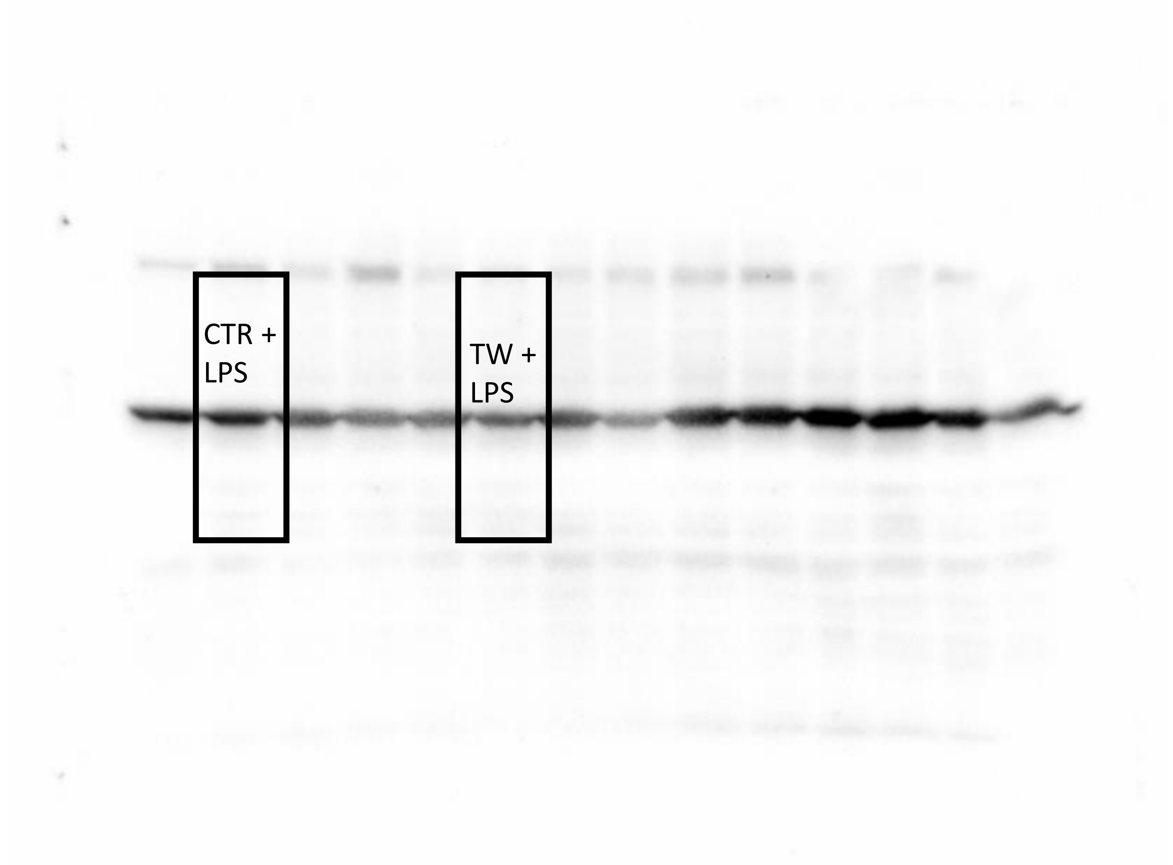

SBS TW 1

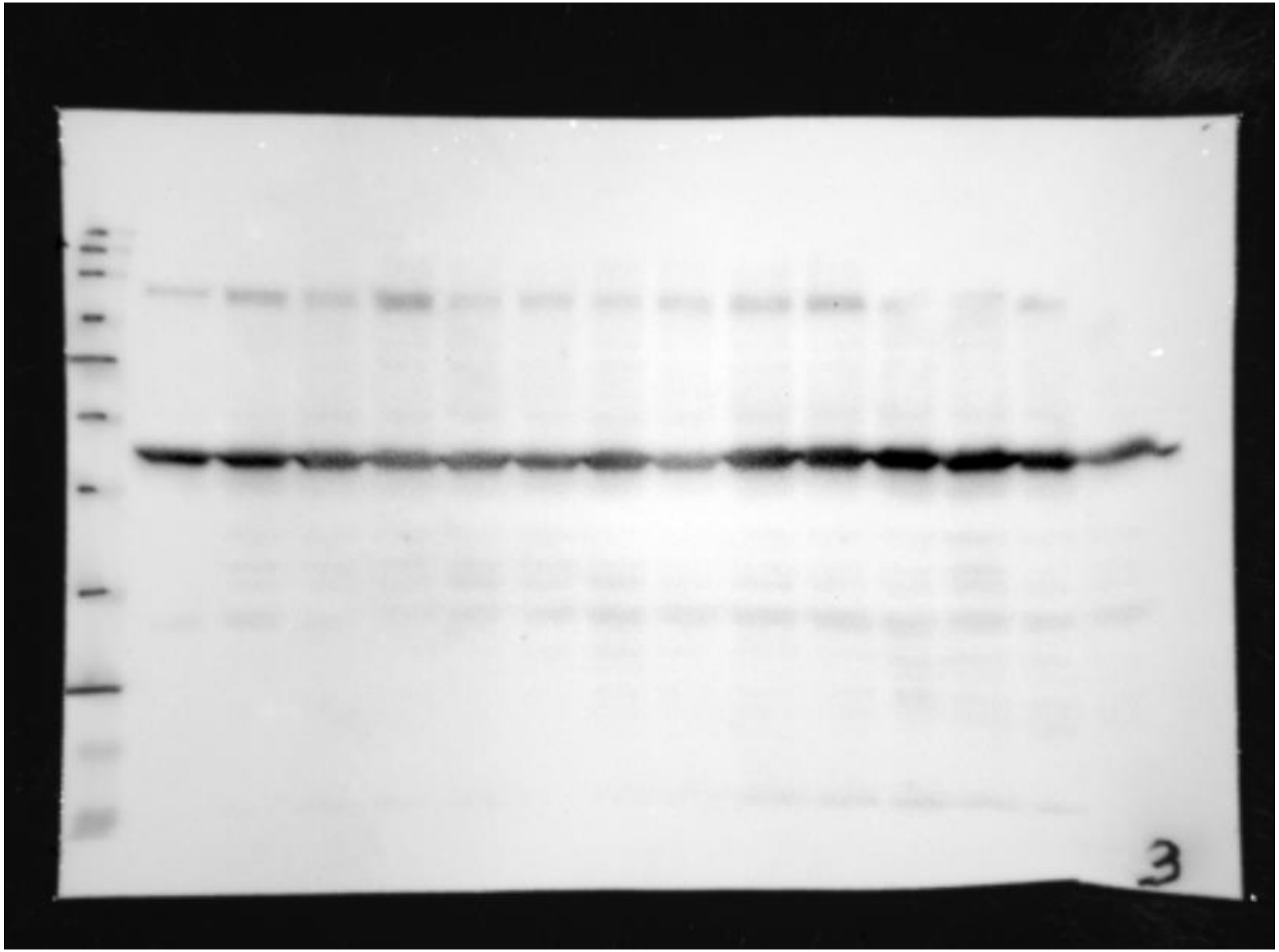

SBS TW 2

INOS

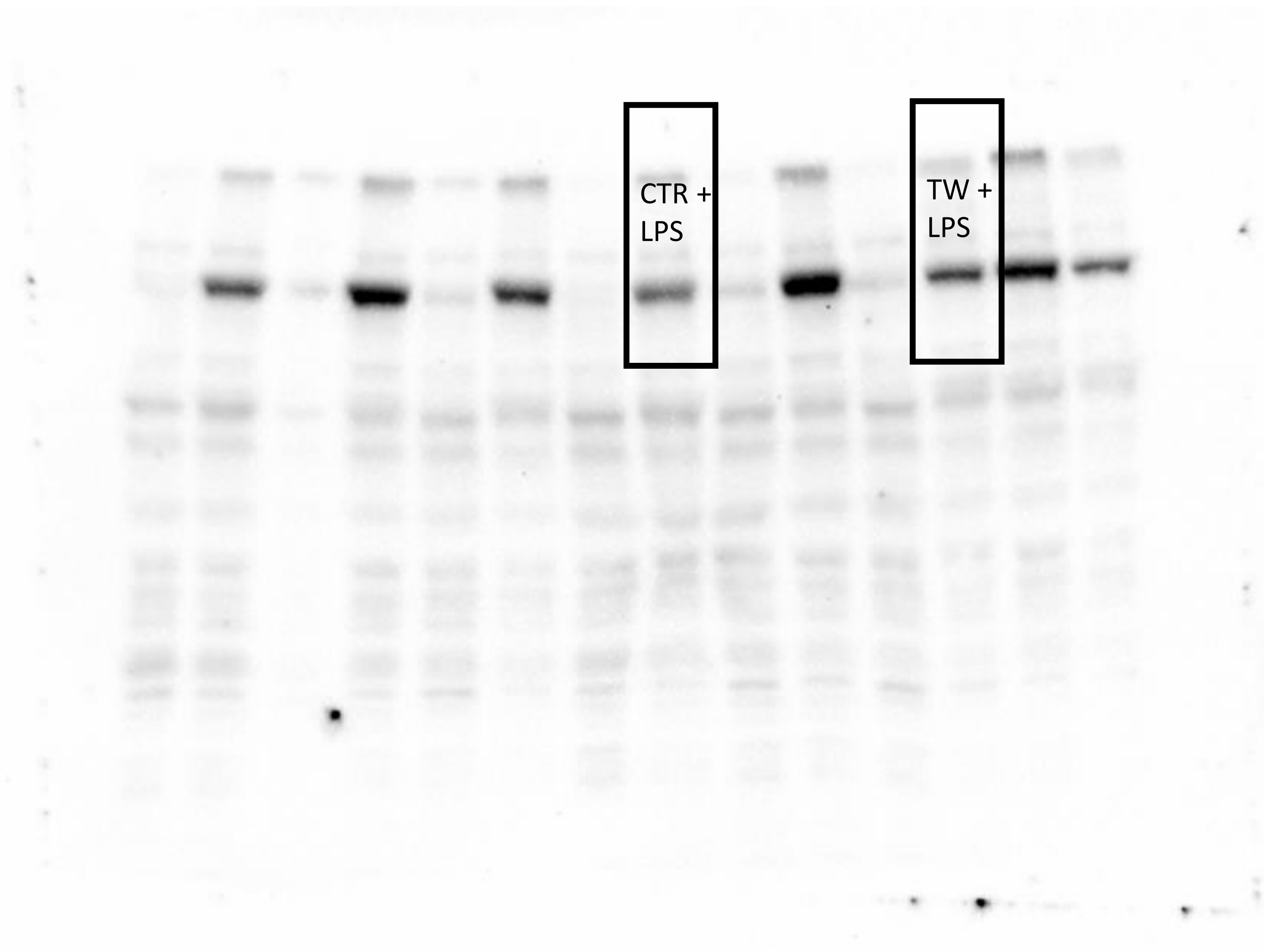

TUB

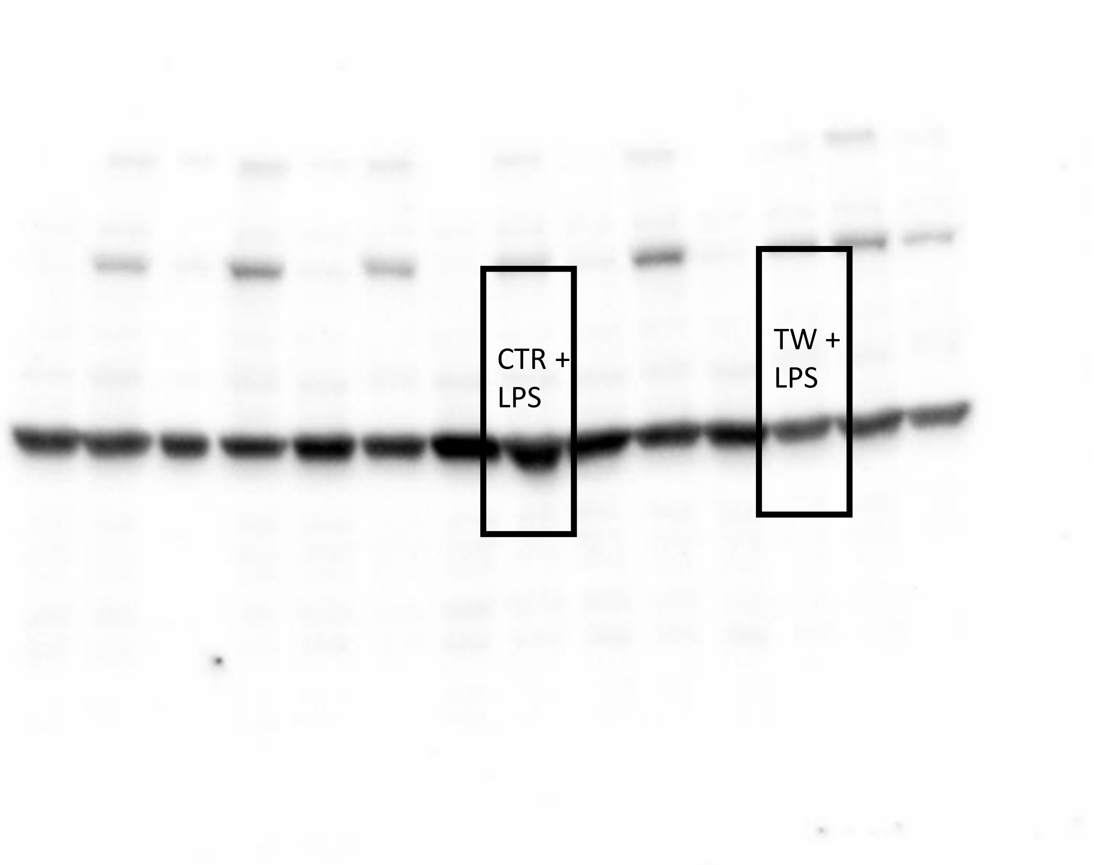

SBS TW 2

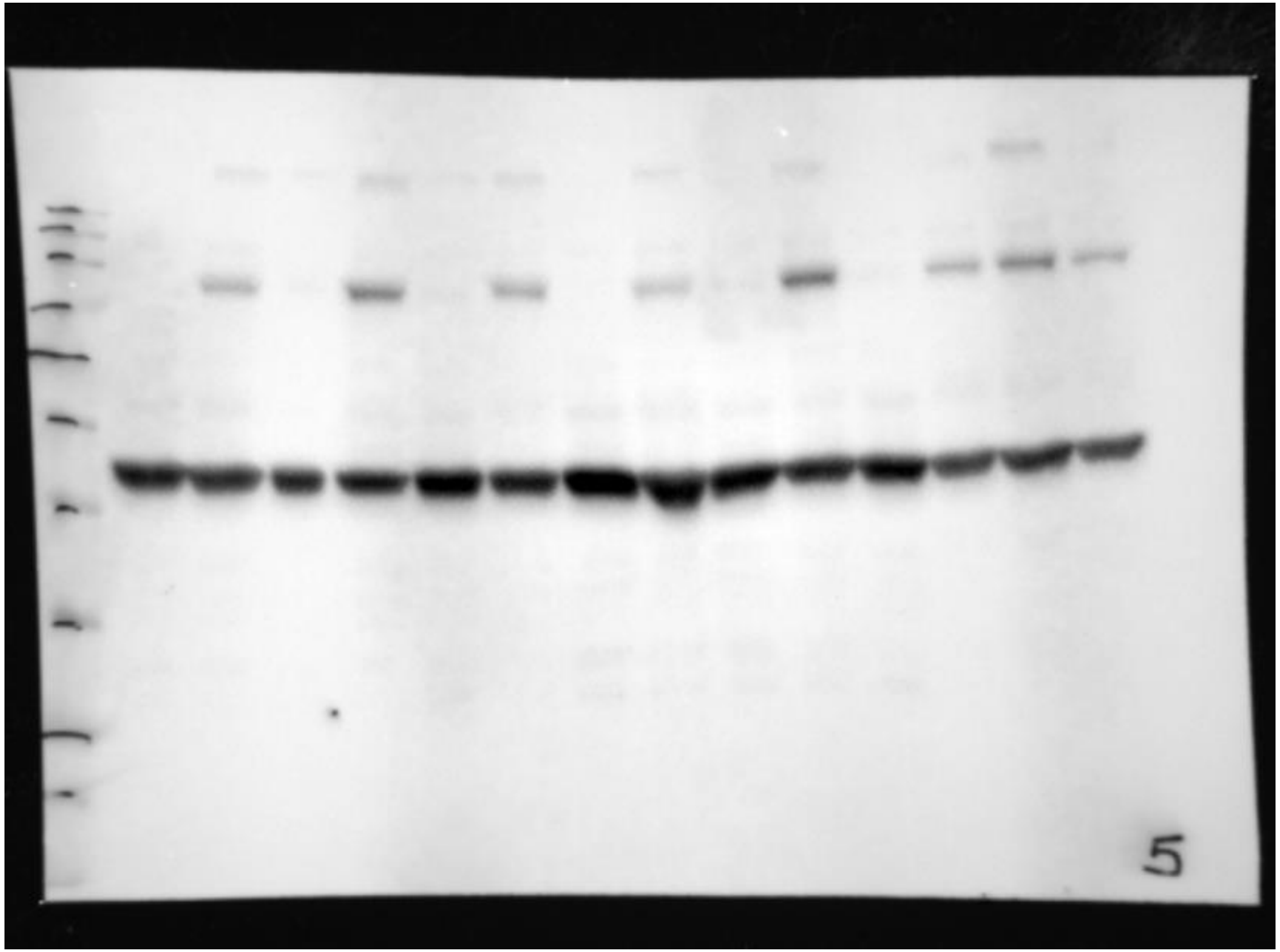

SBS TW 3

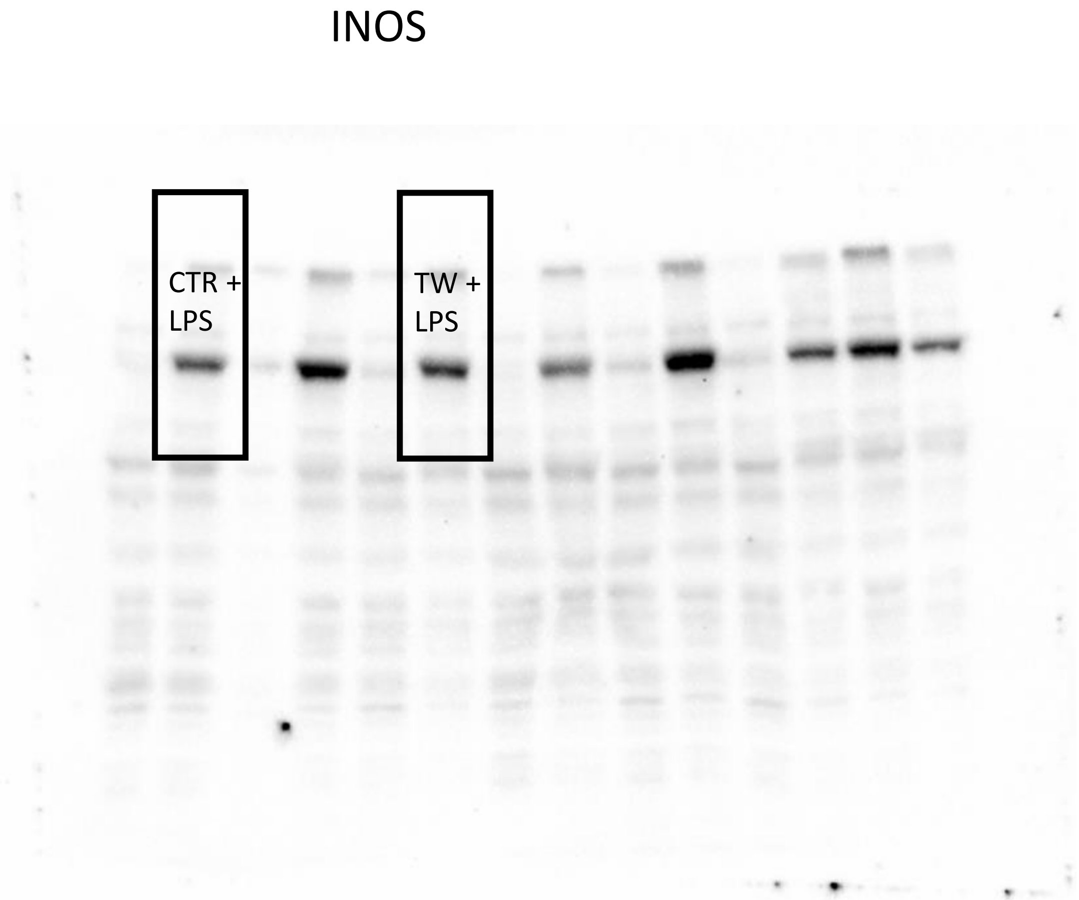

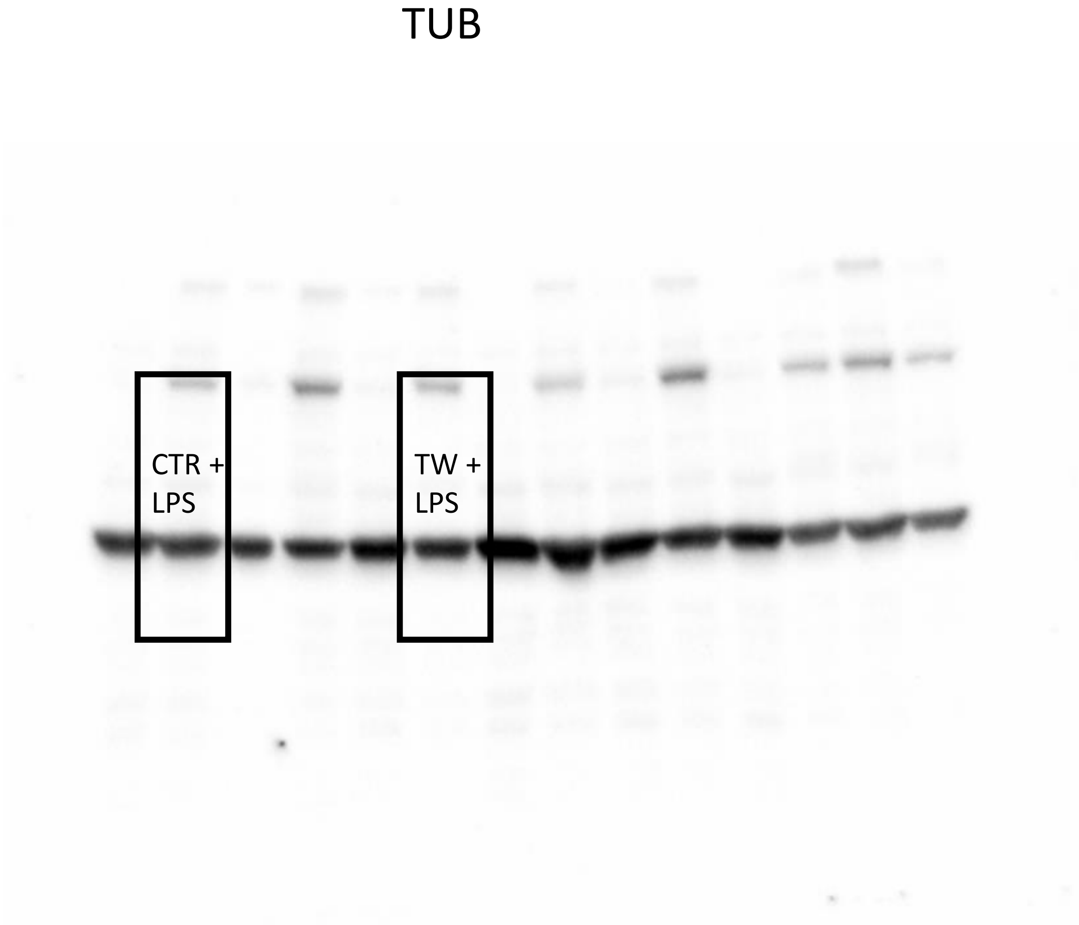

SBS TW 3

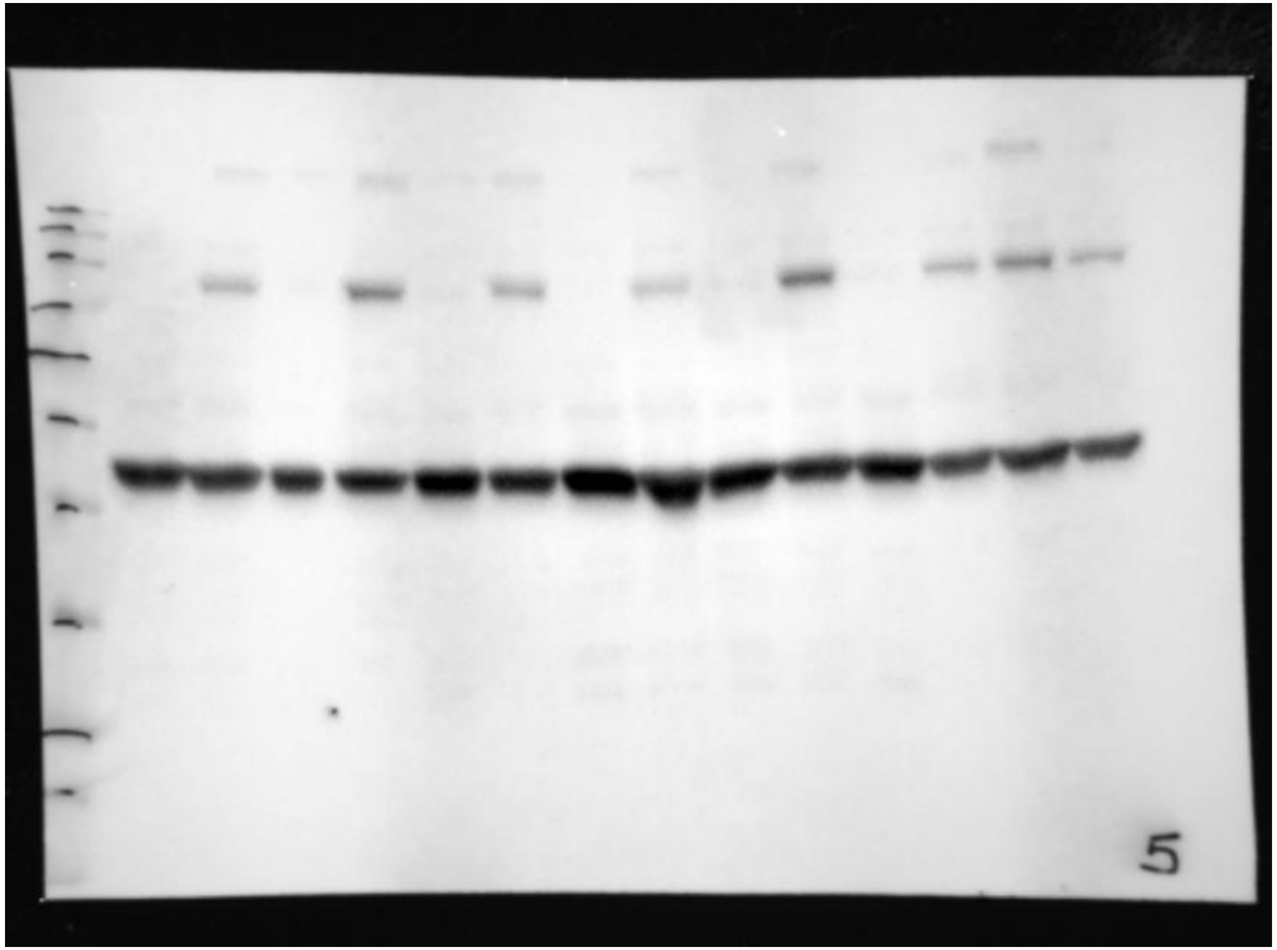

SBS TW 4

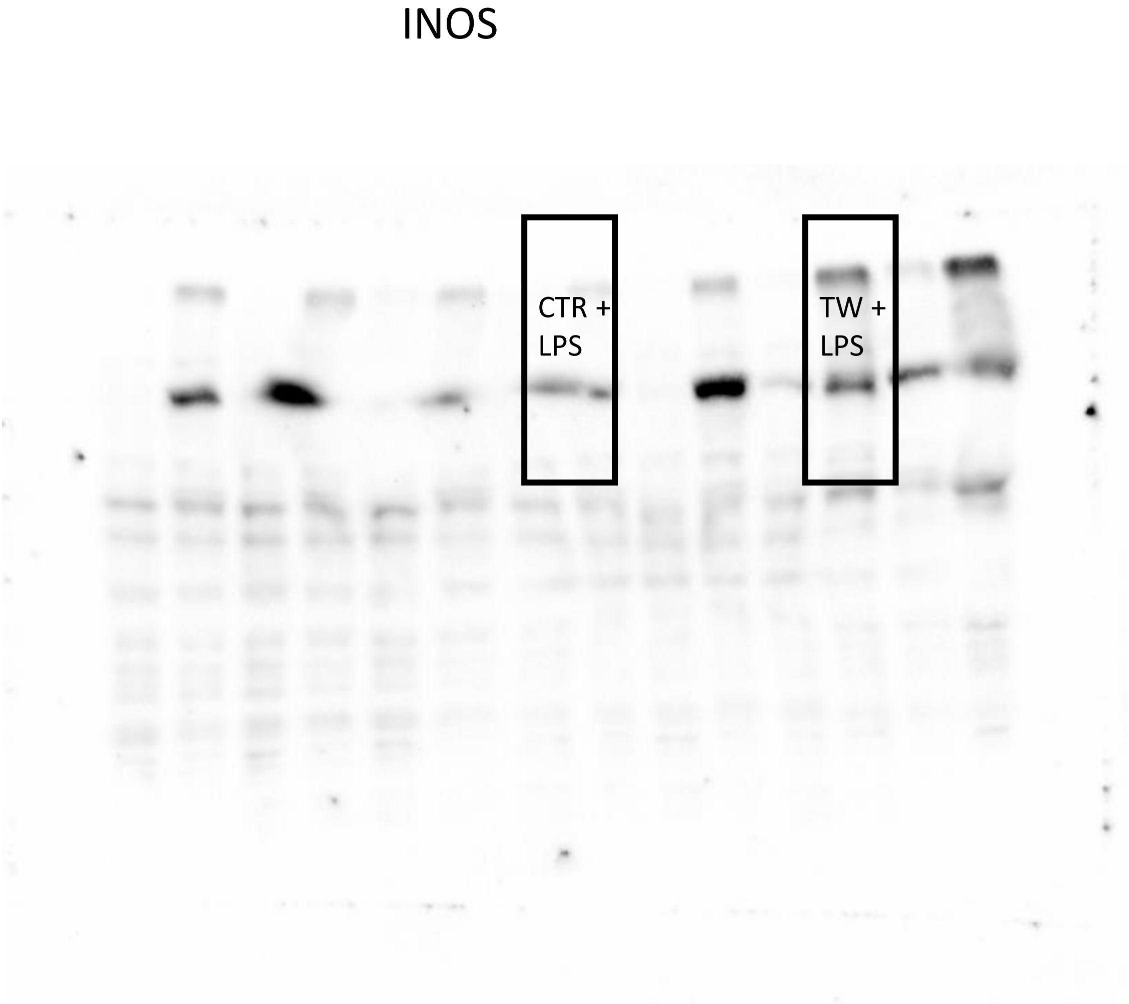

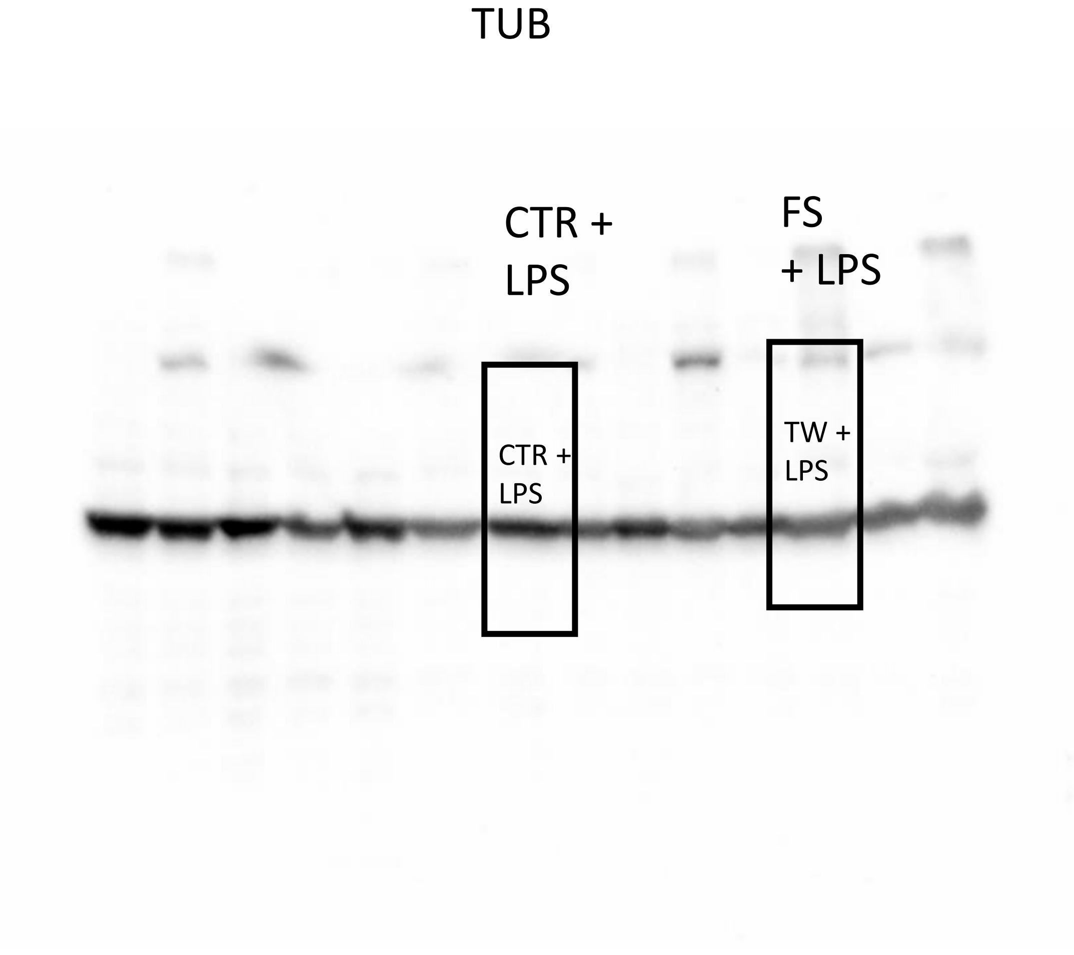

SBS TW 4

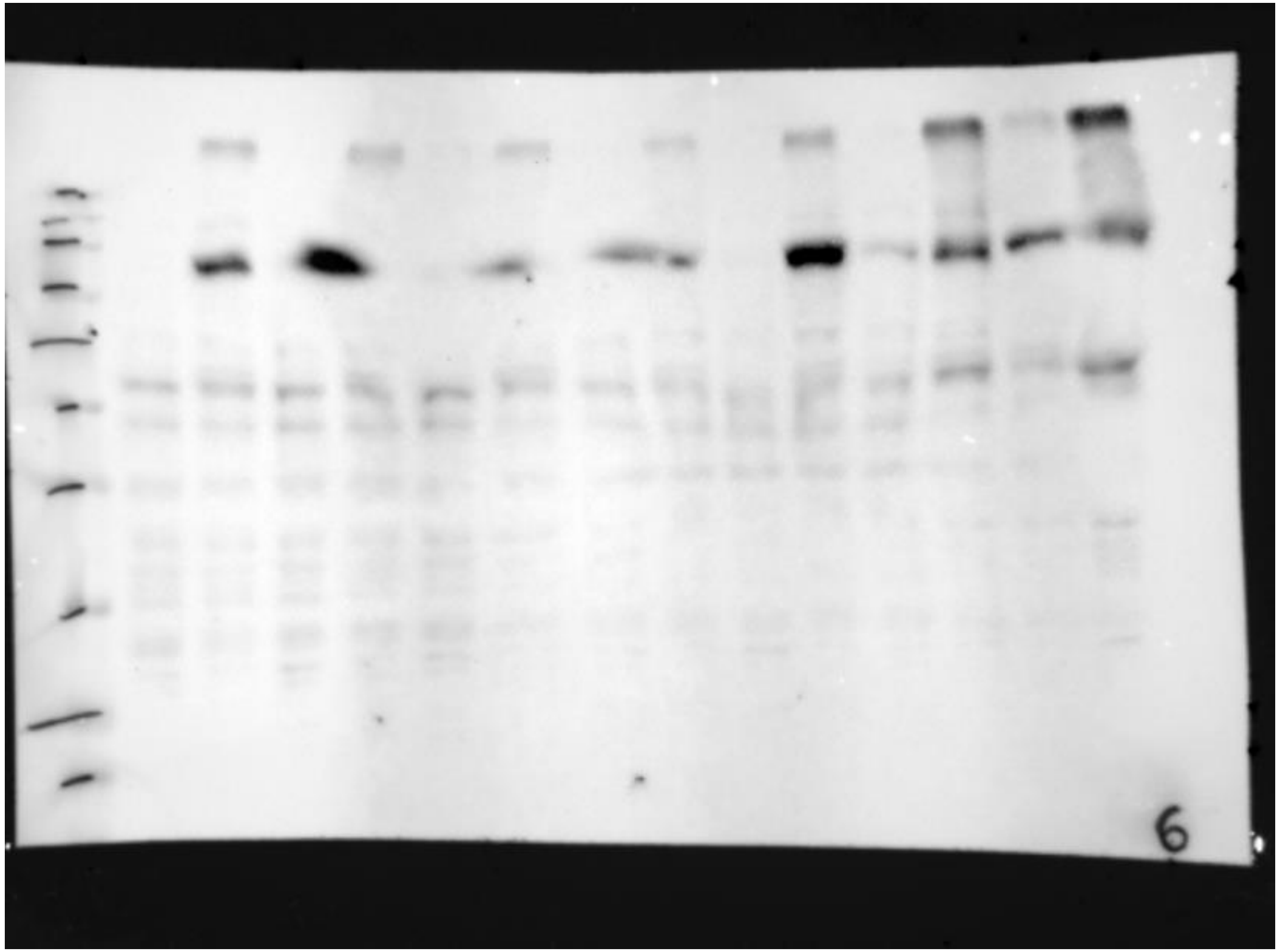

SBS TW 5

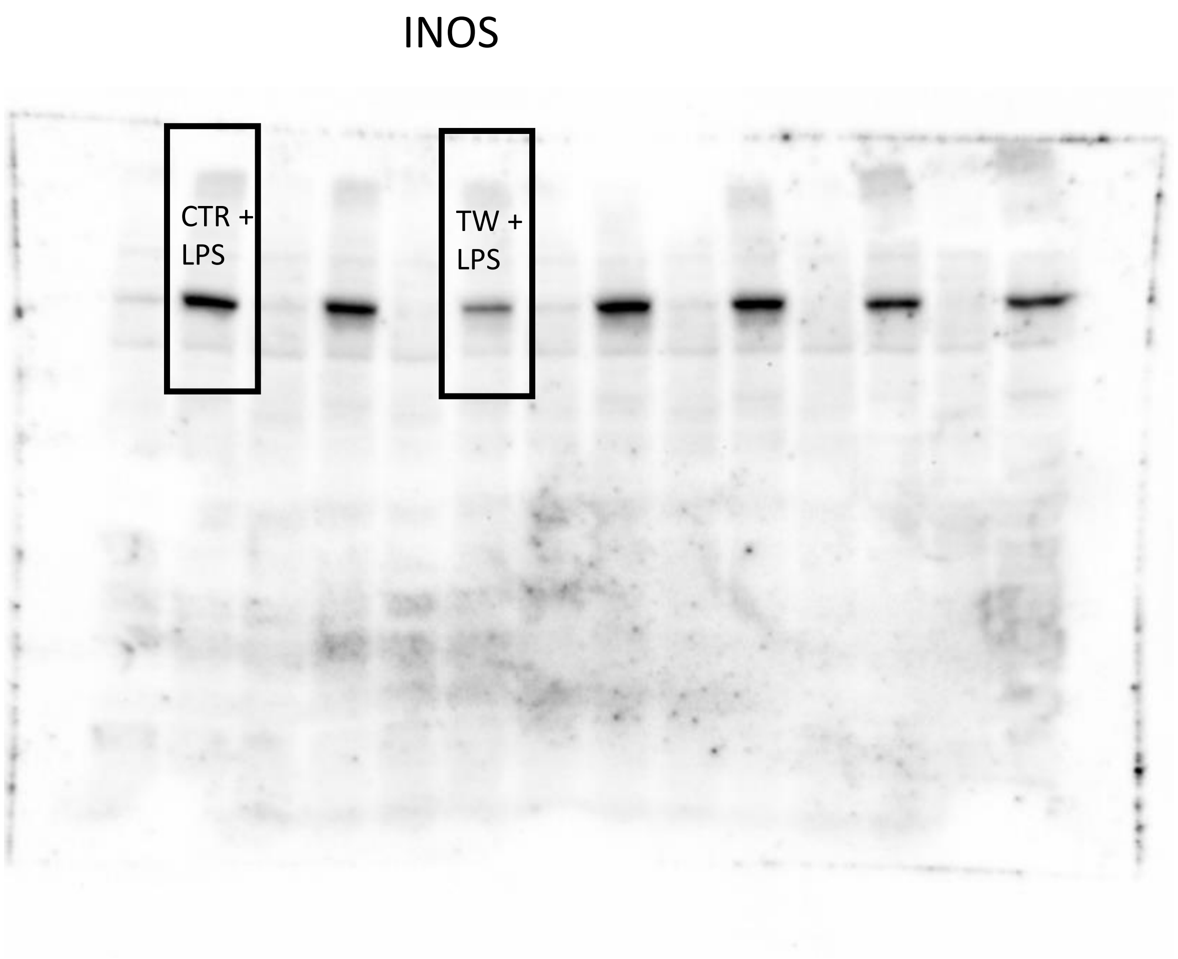

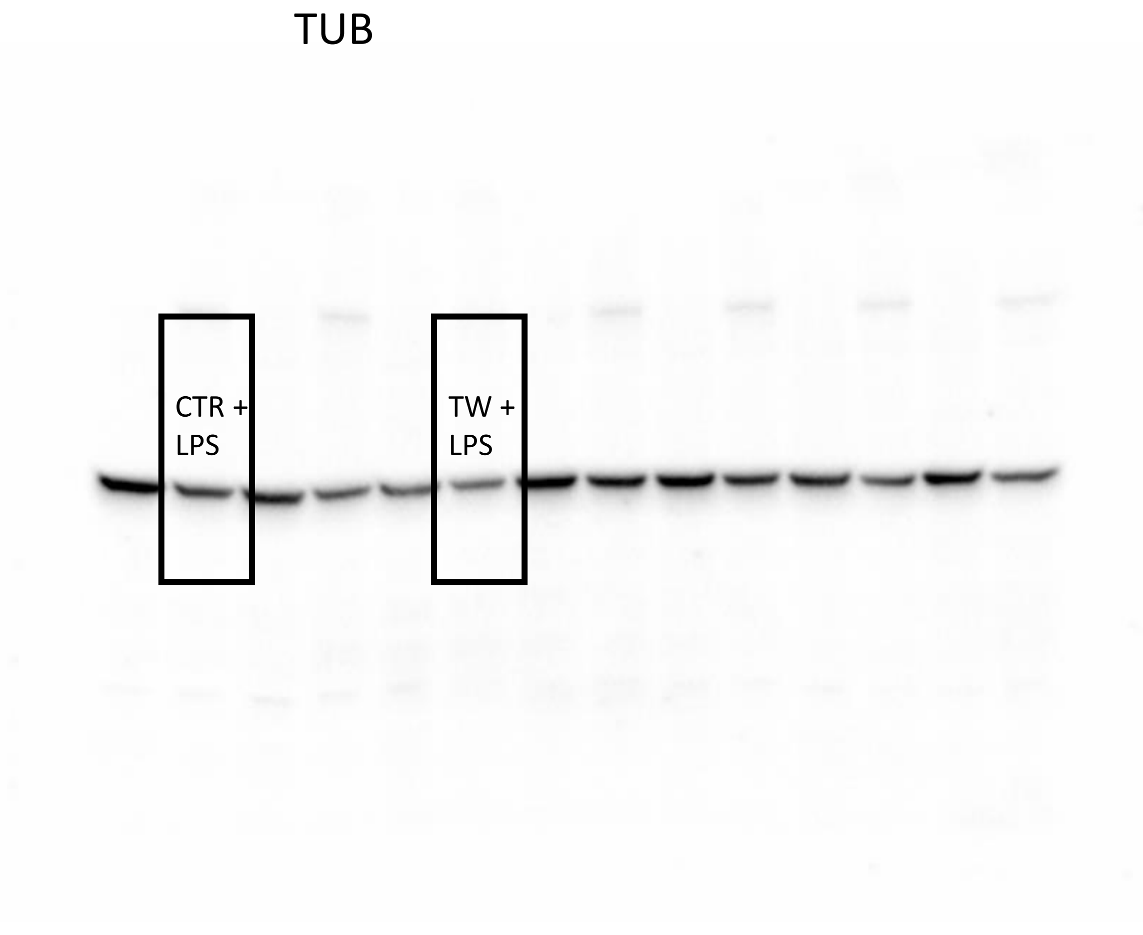

SBS TW 5

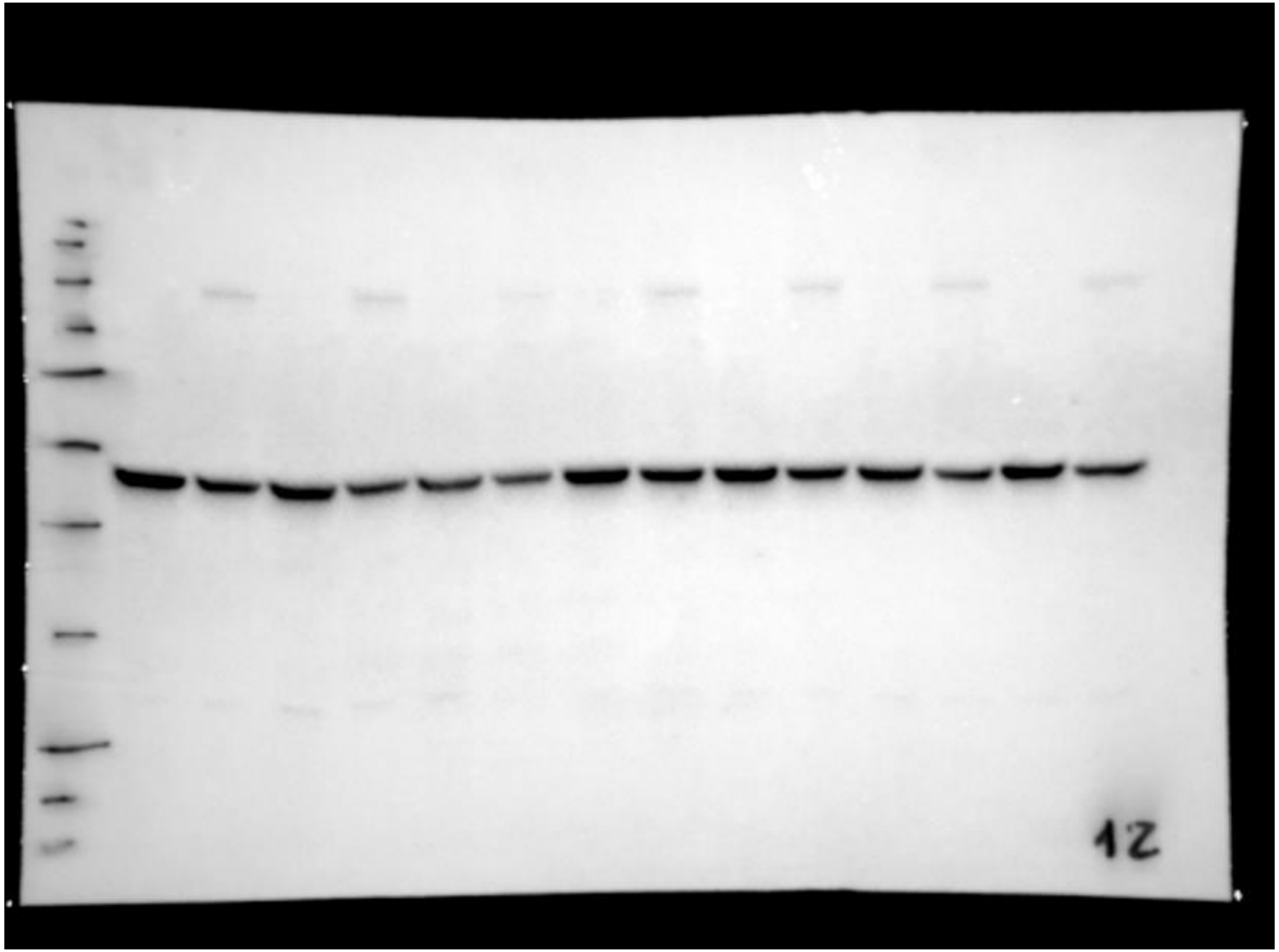

SBS TW 6

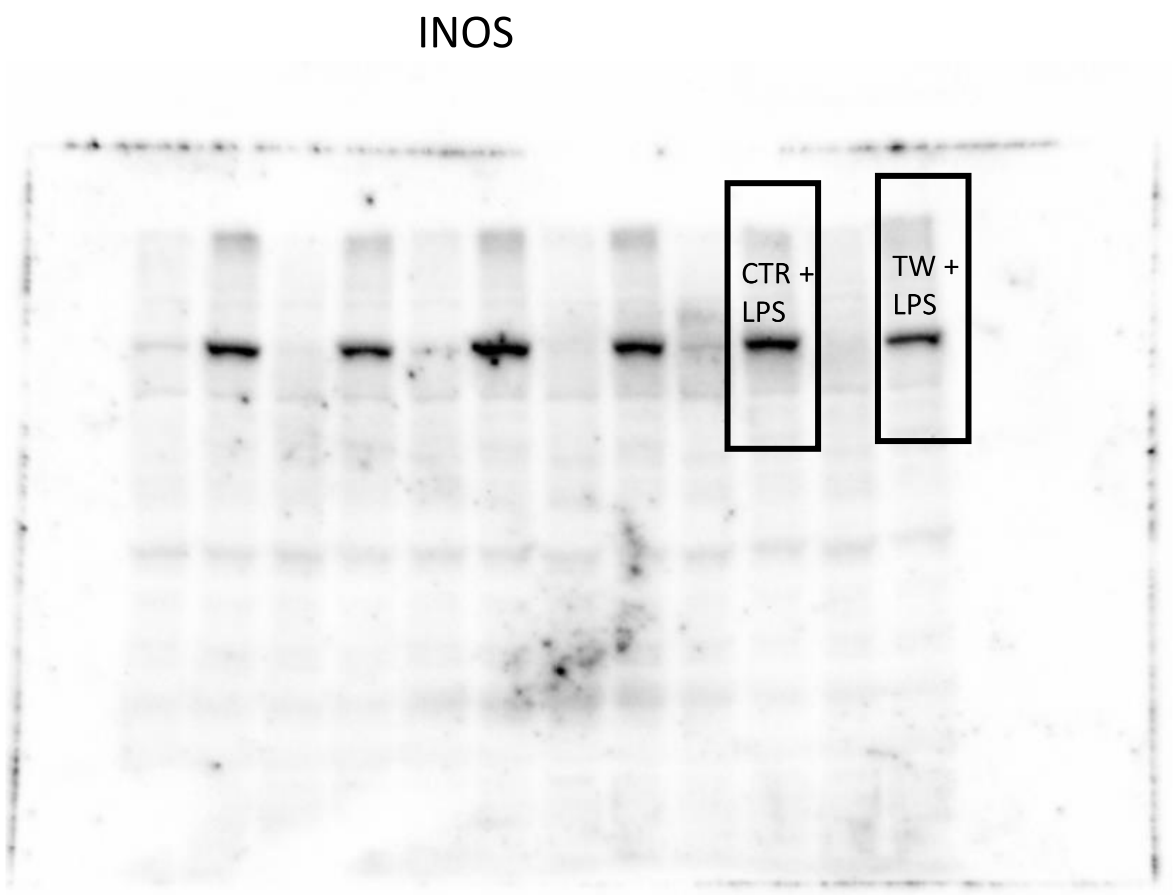

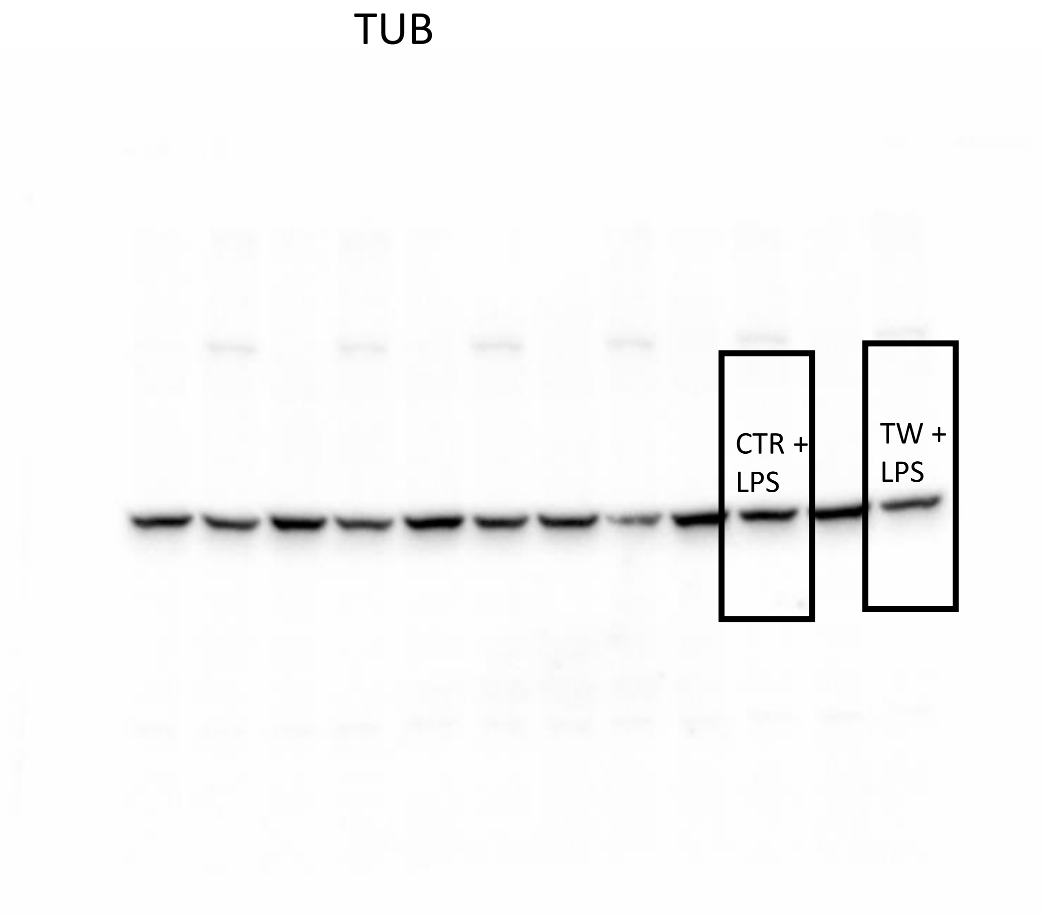

SBS TW 6

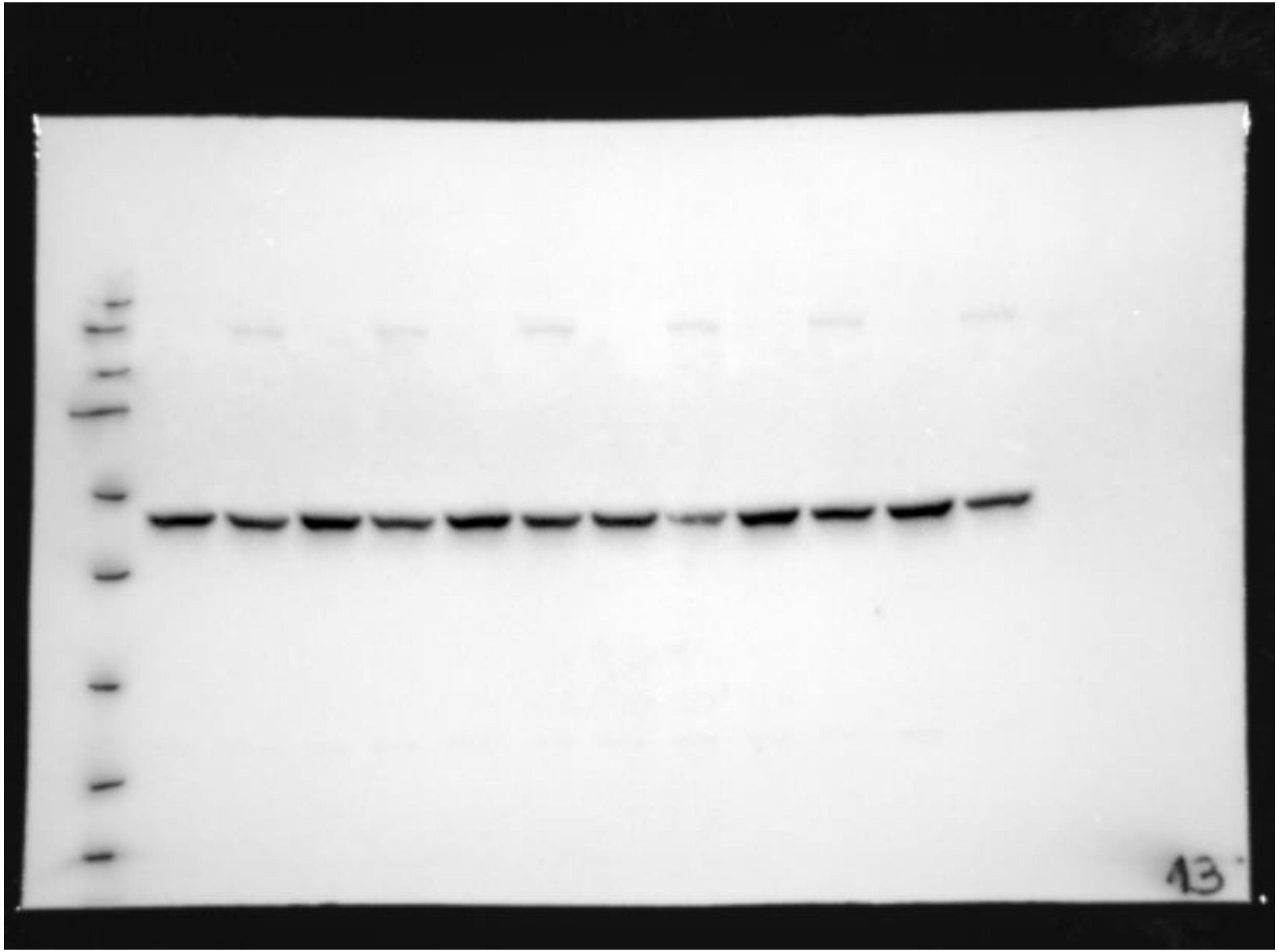

SBS TW 7

INOS

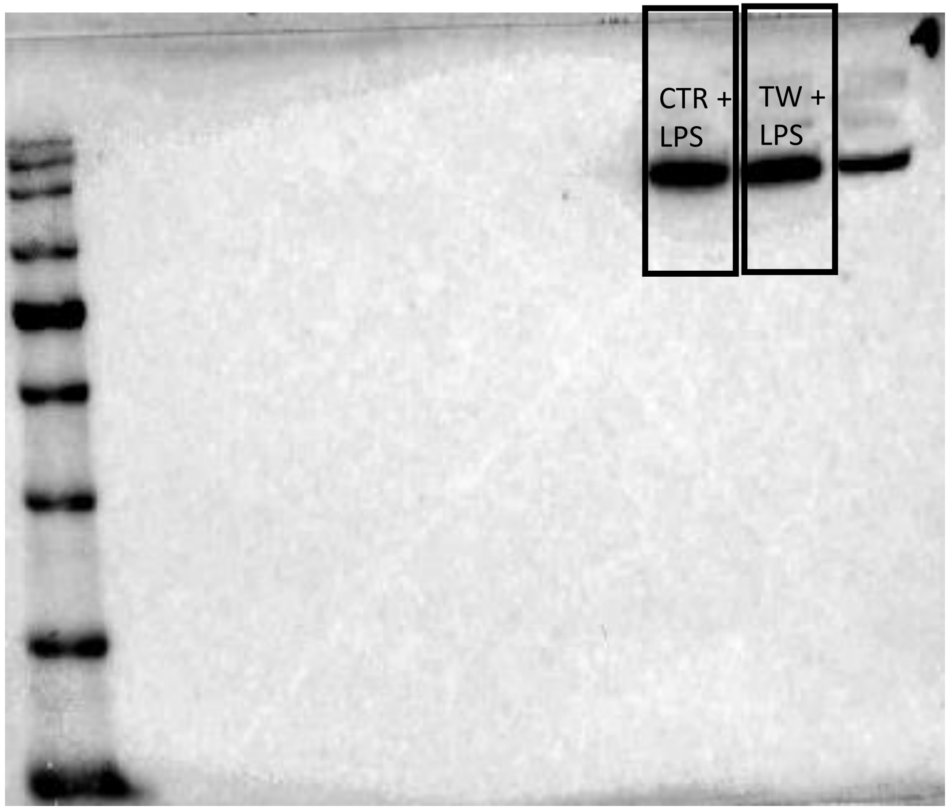

TUB

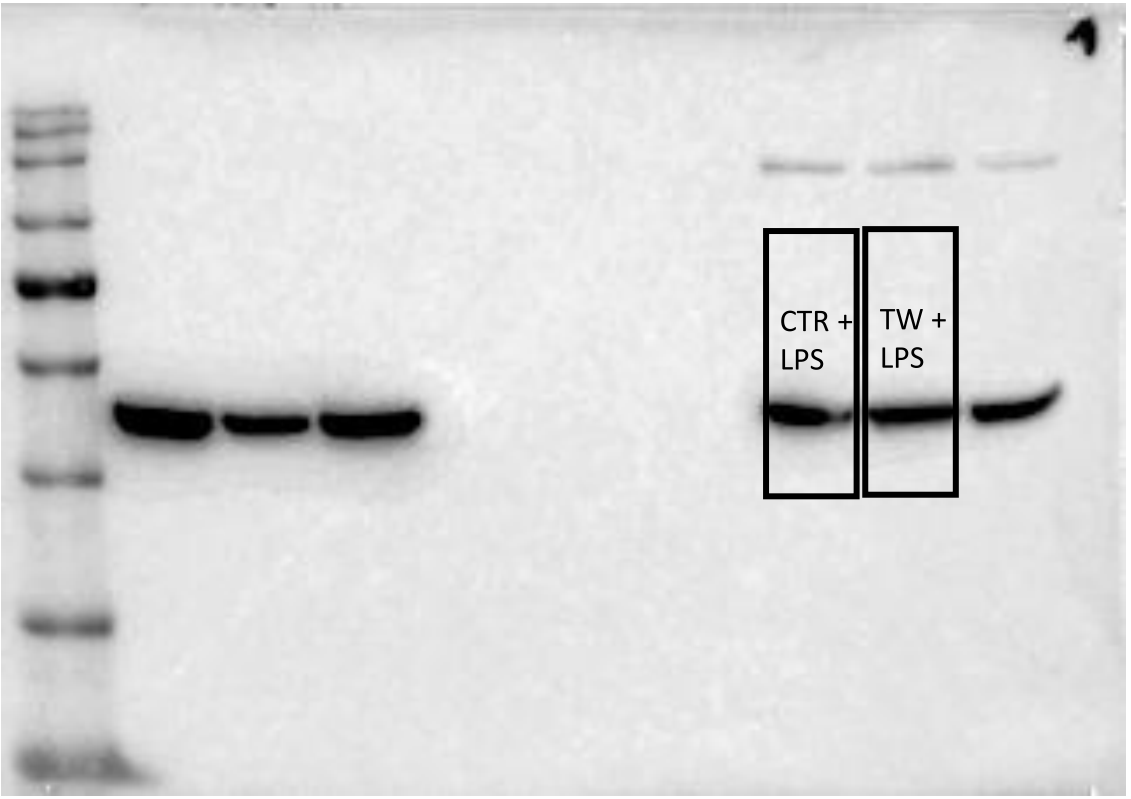

SBSF TW 1

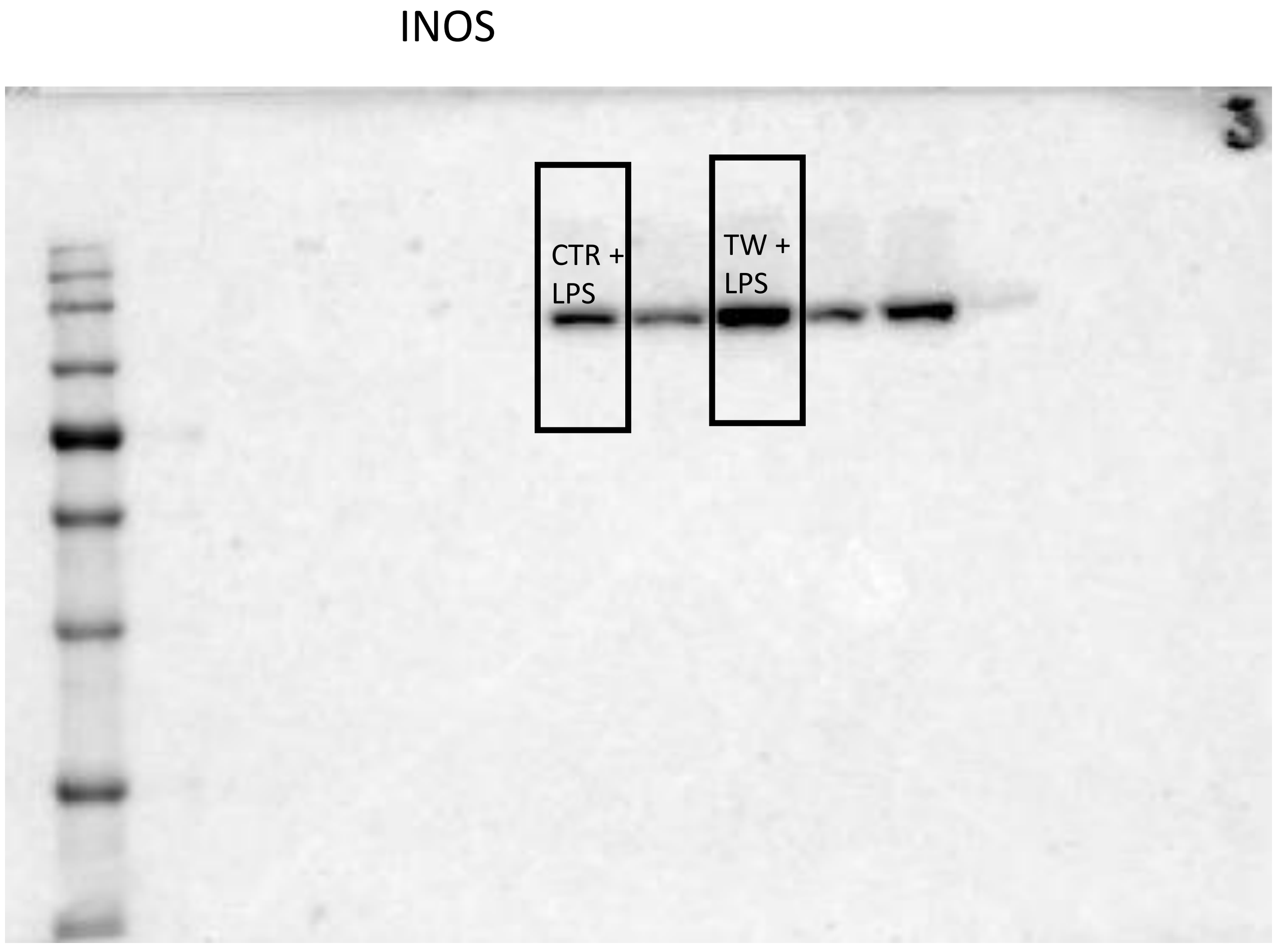

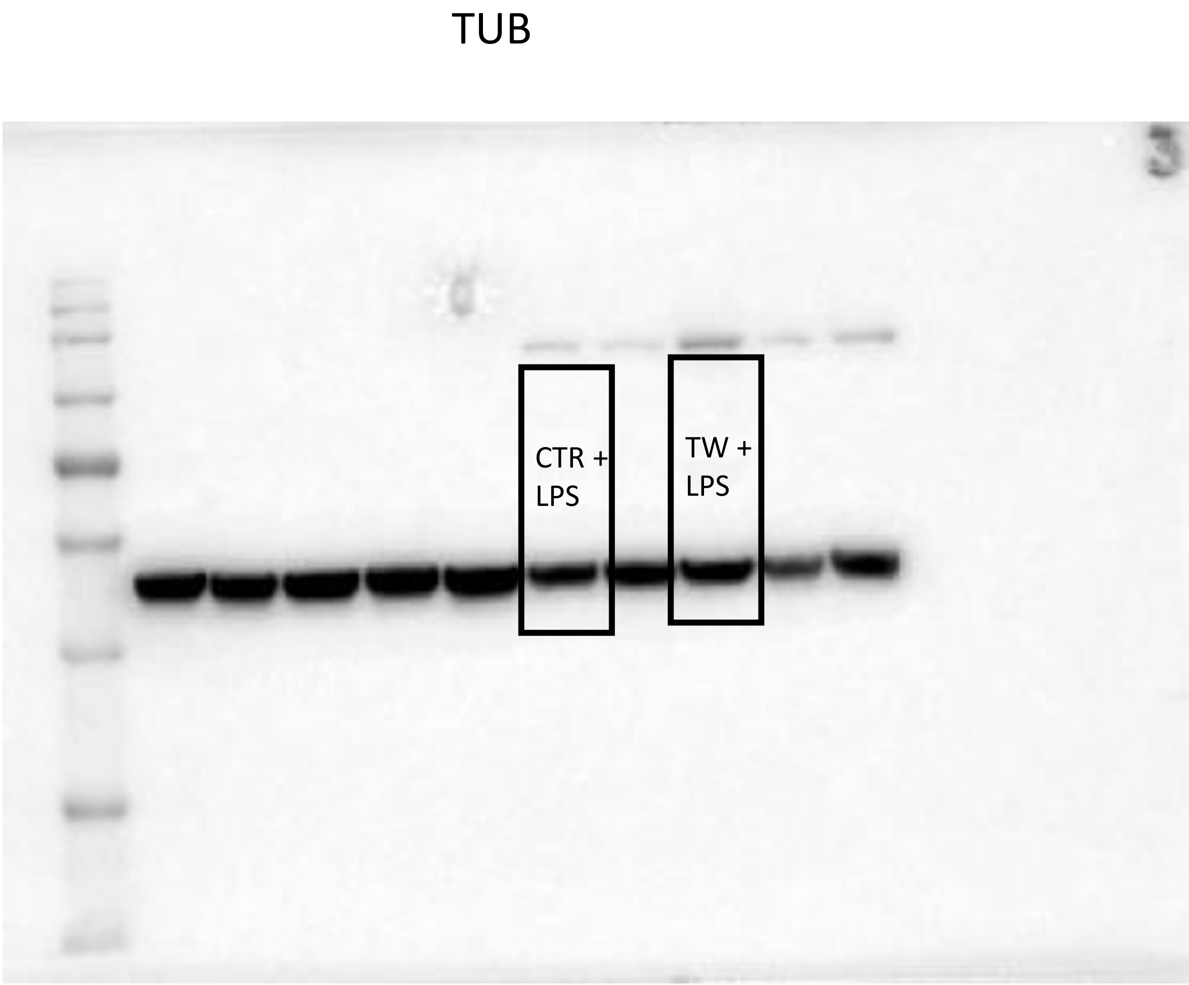

SBSF TW 2

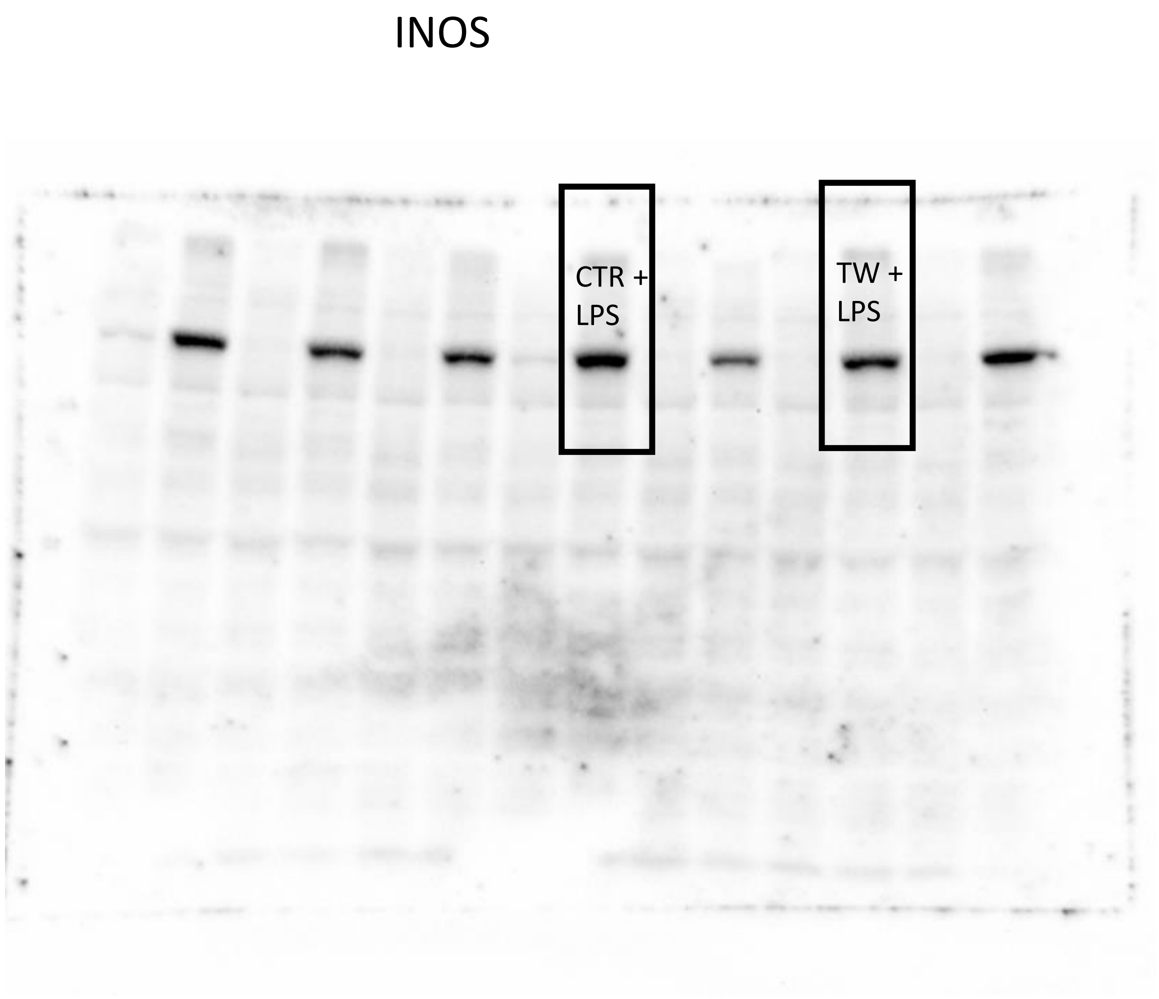

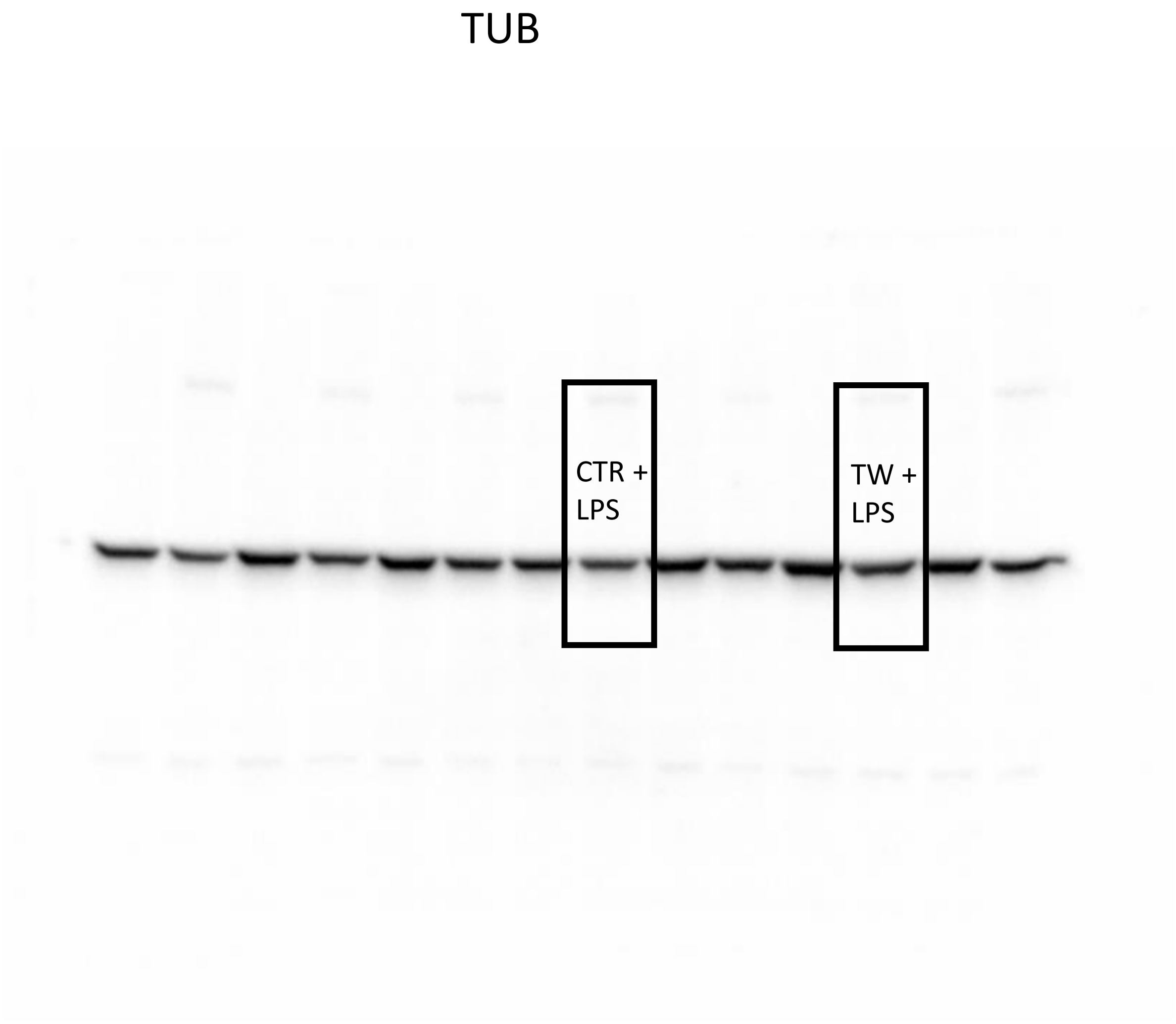

SBSF TW 2

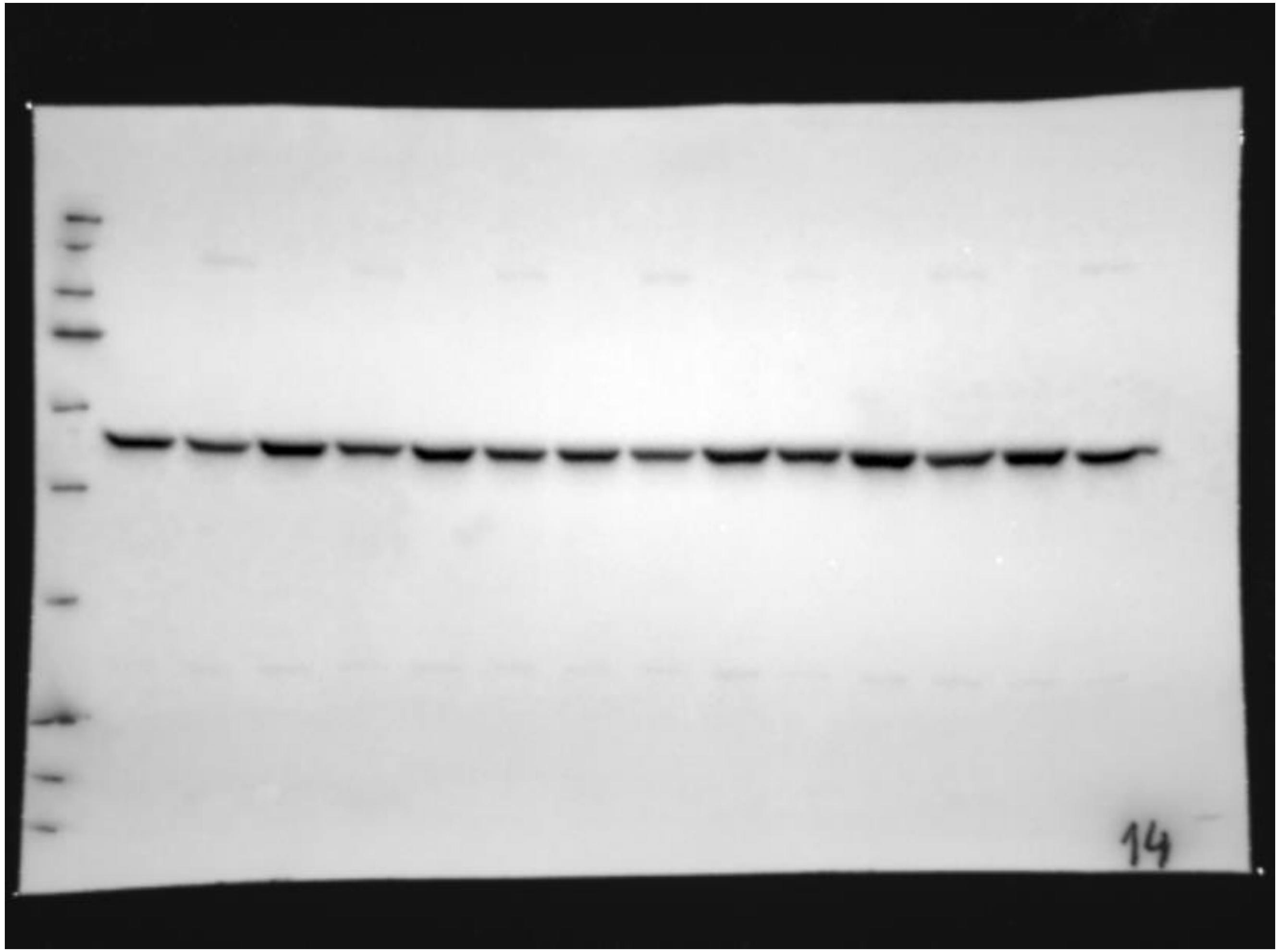

SBSF TW 3

INOS

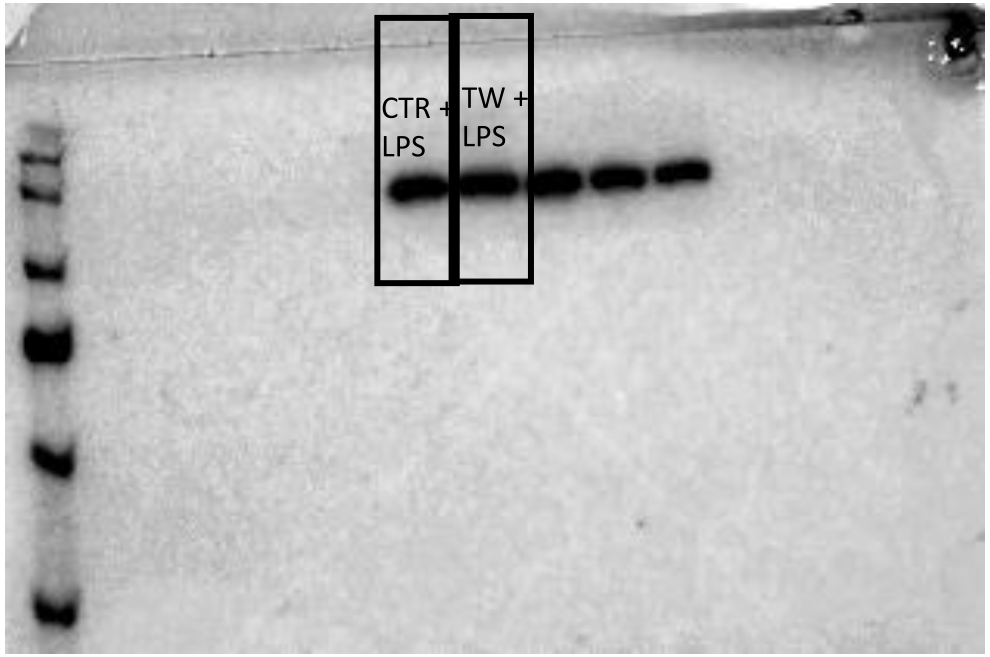

TUB

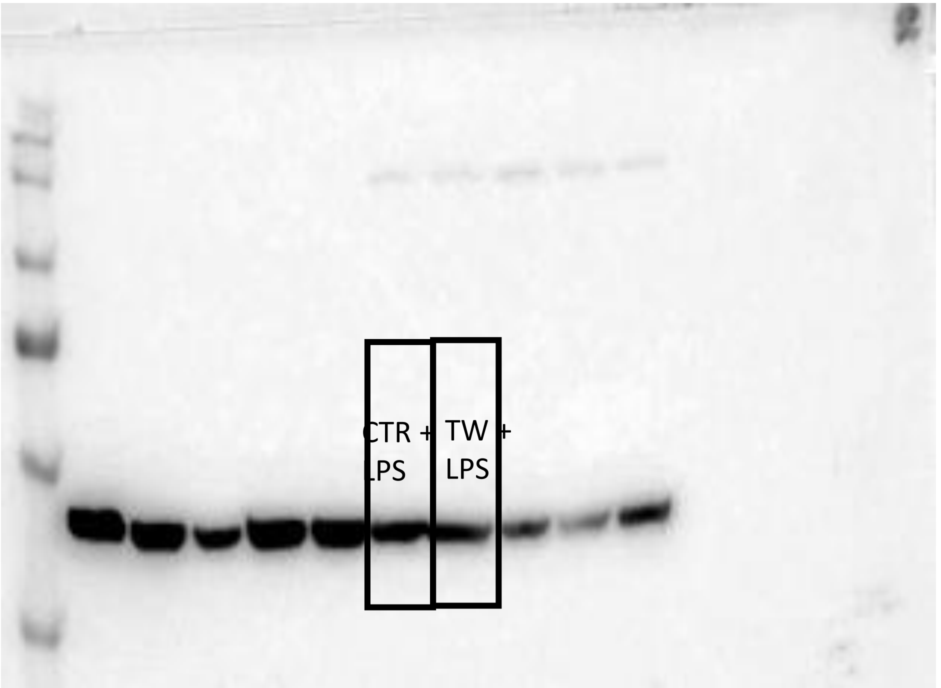

SCS TW

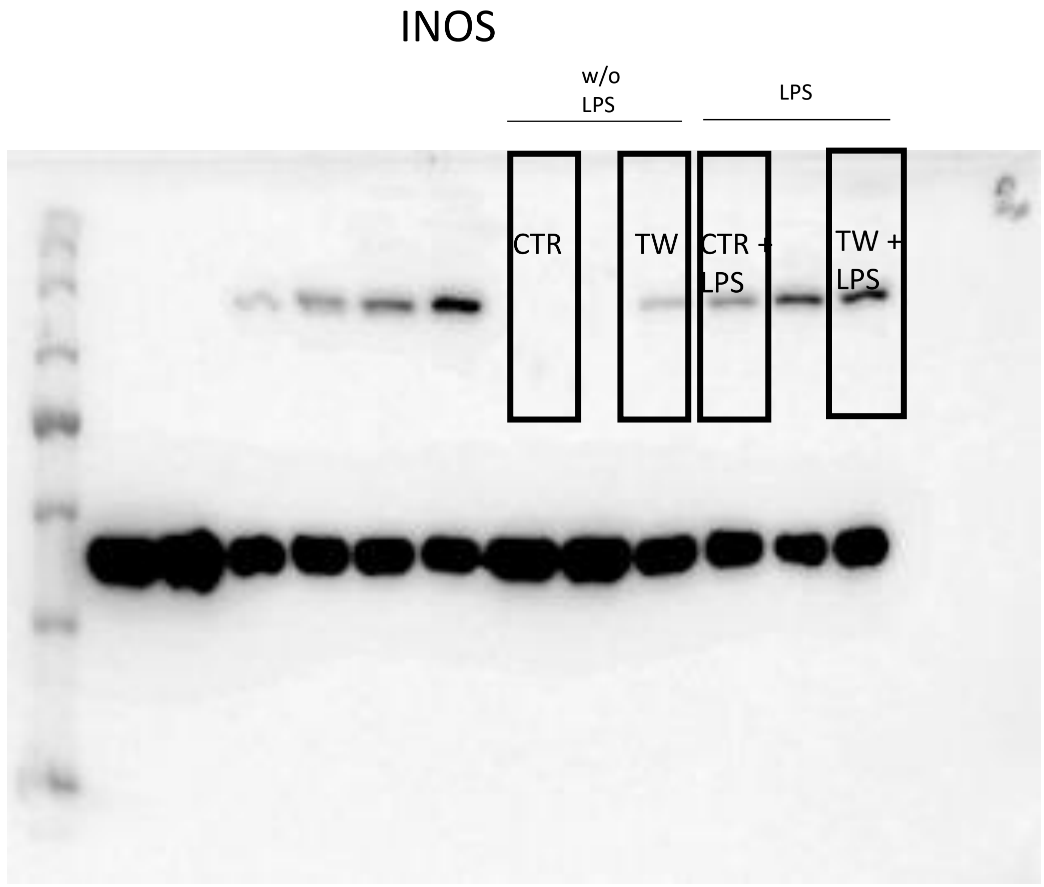
INOS
w/o
LPS
LPS
CTR
TW
CTR +
LPS
TW +
LPS

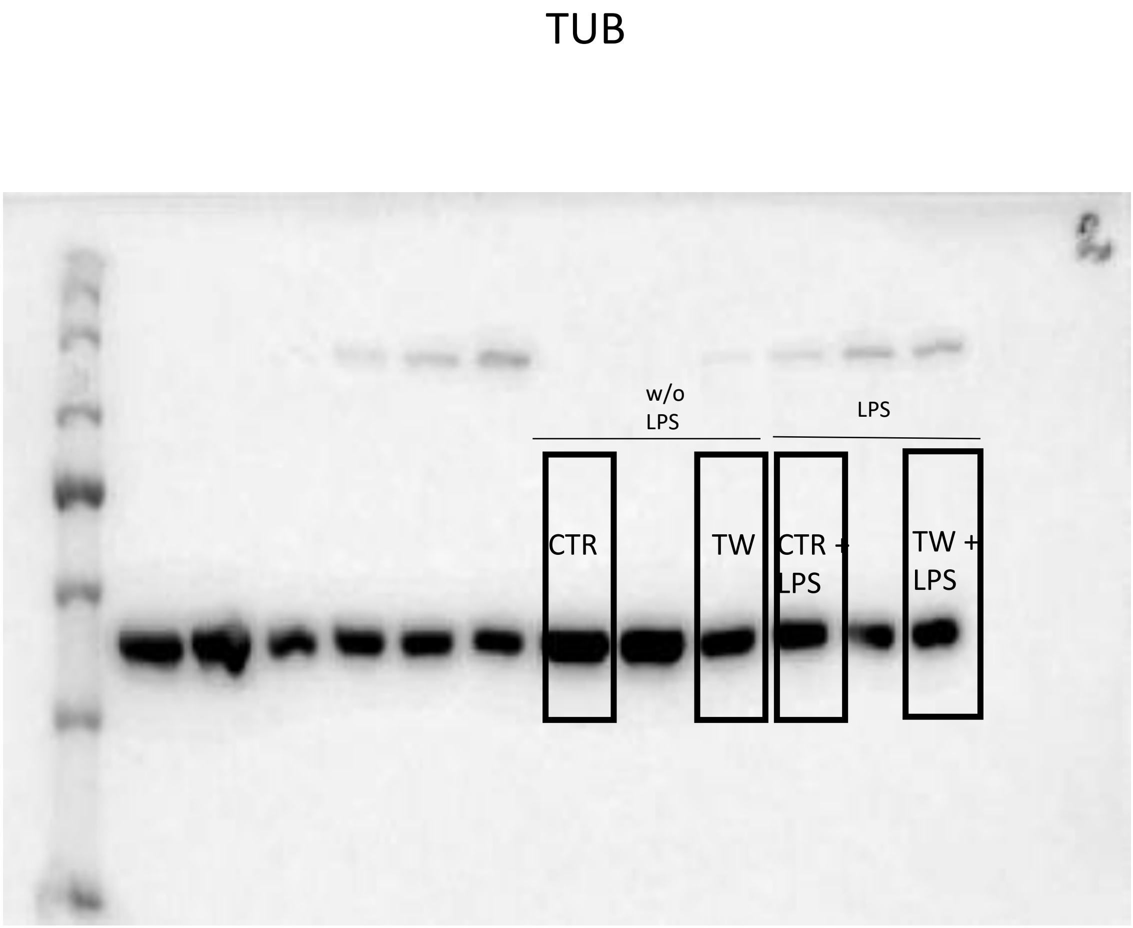
TUB
w/o
LPS
LPS
CTR
TW
CTR +
LPS
TW +
LPS

CS TW

INOS

TUB

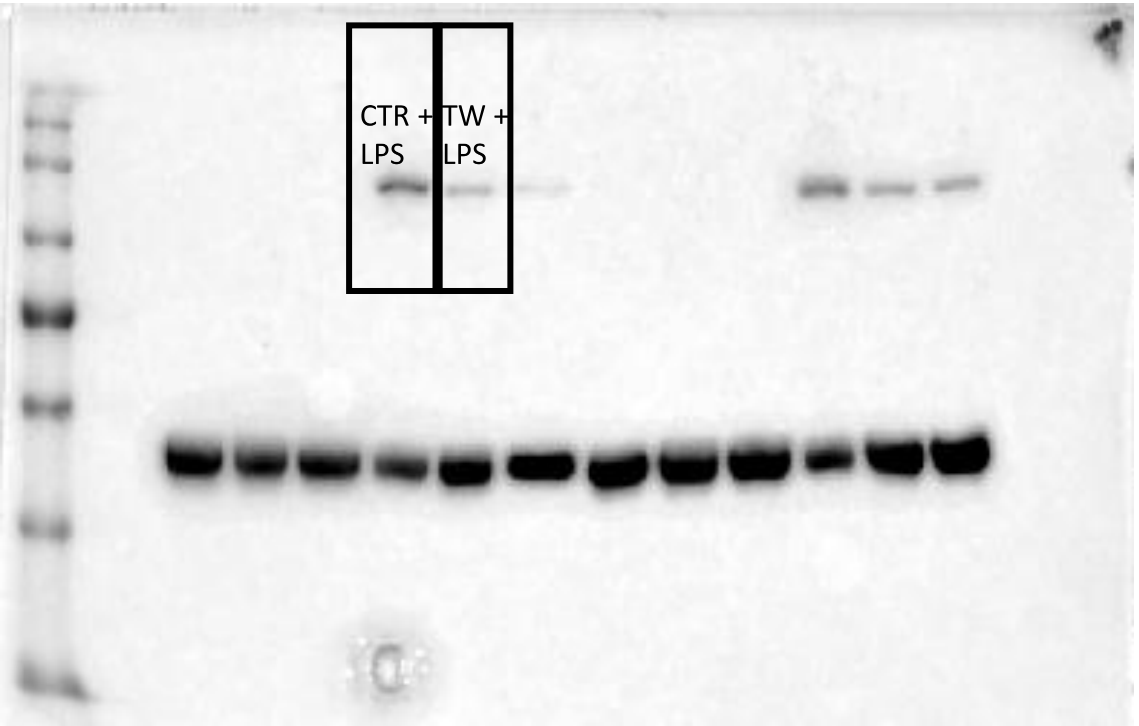

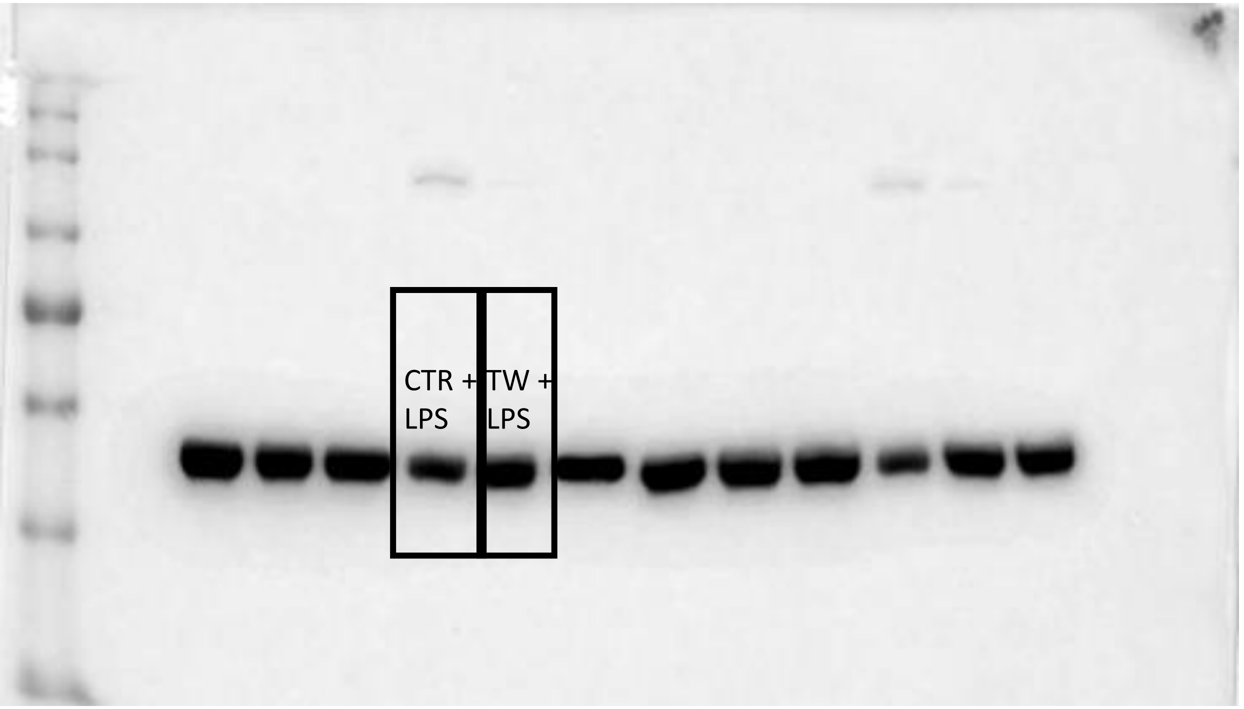

SBSF TW BMF

INOS

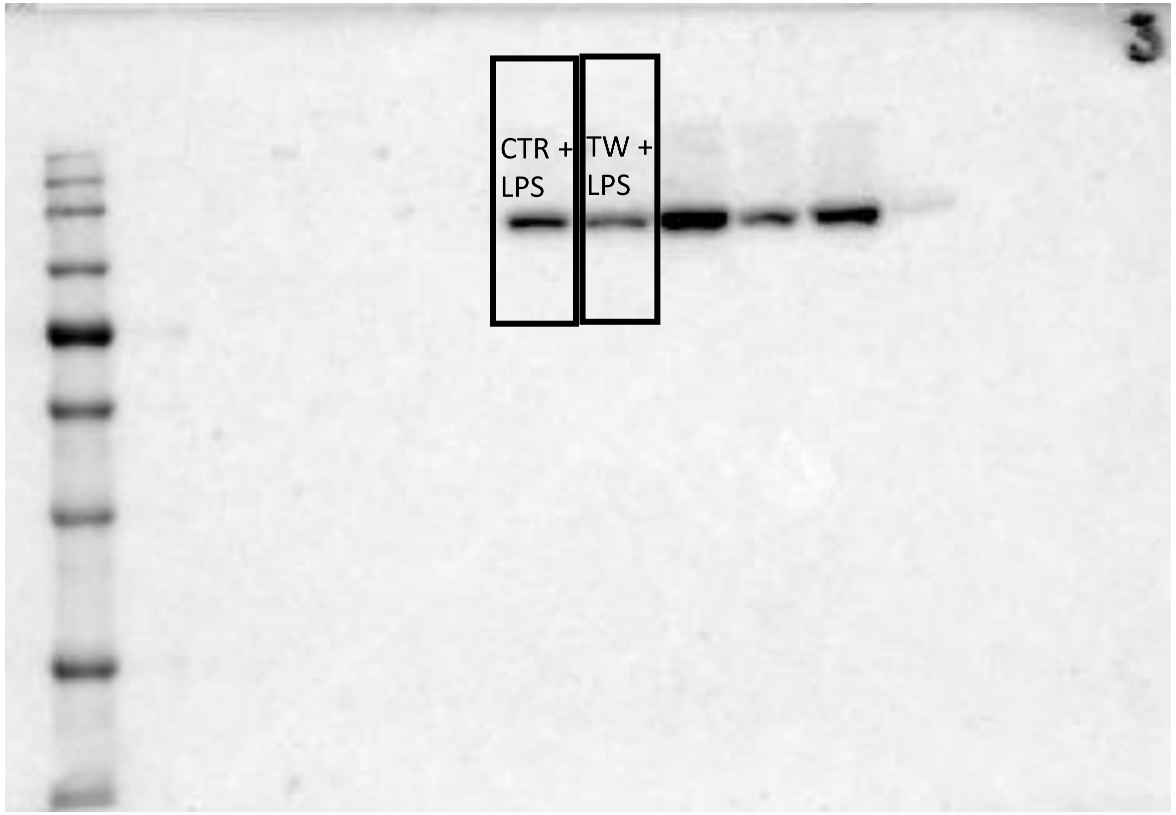

TUB

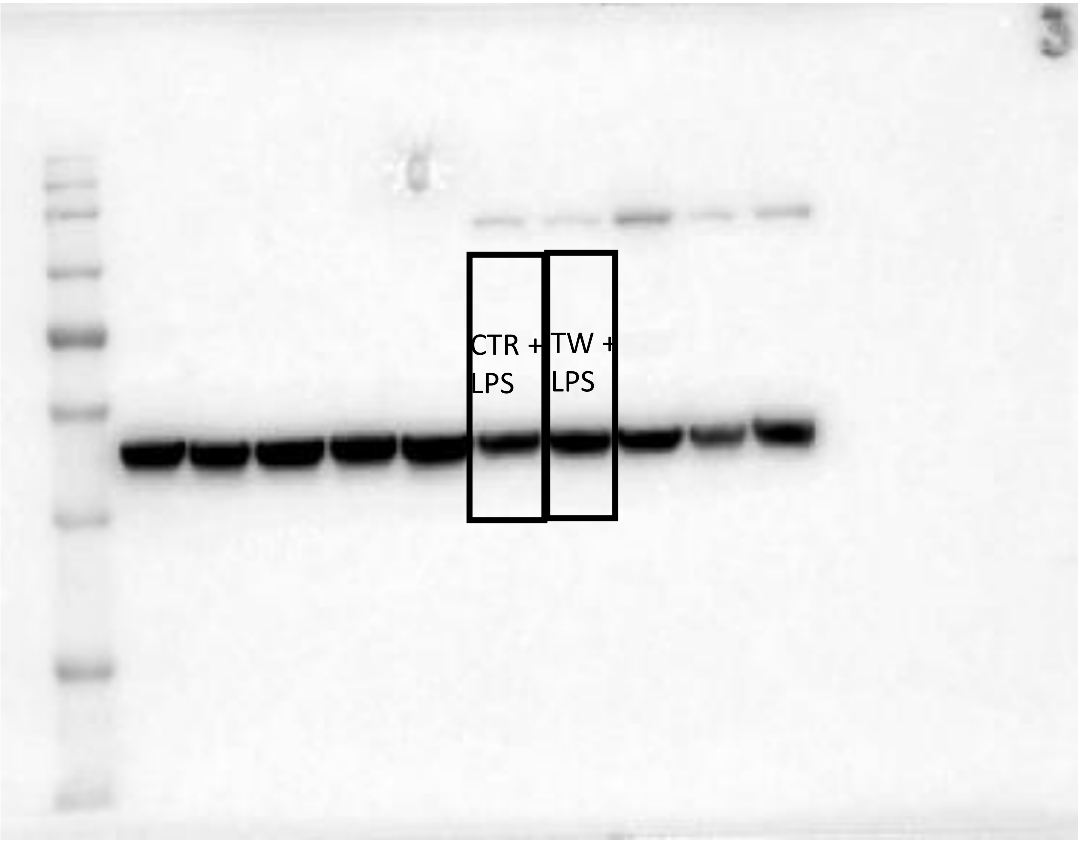

SC TW

INOS

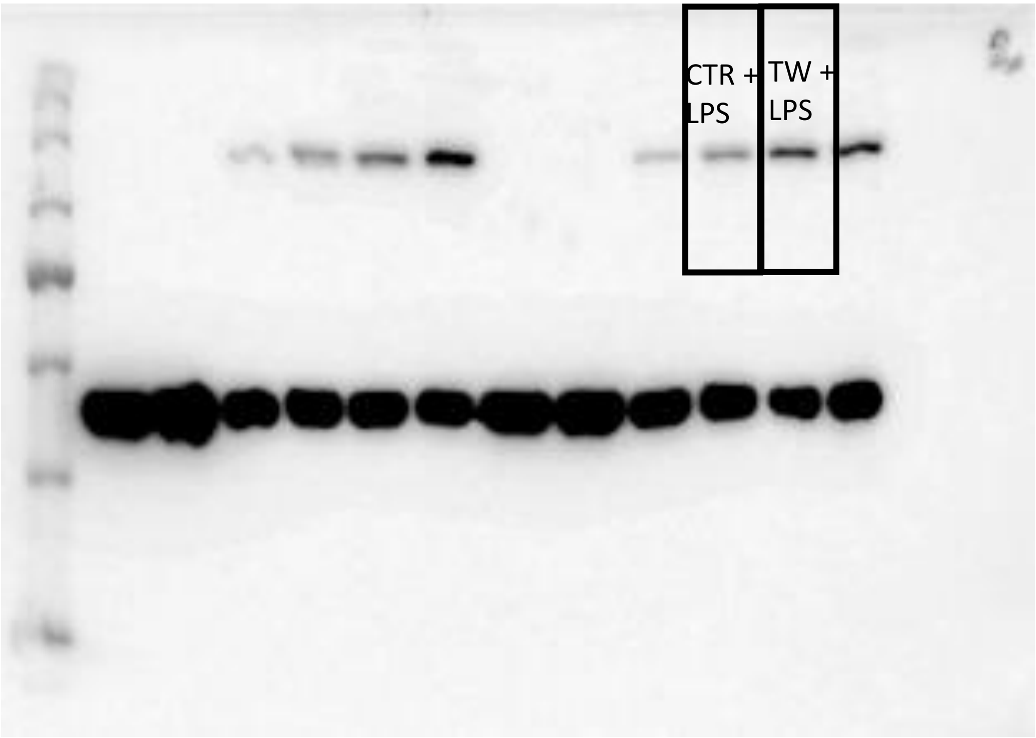

TUB

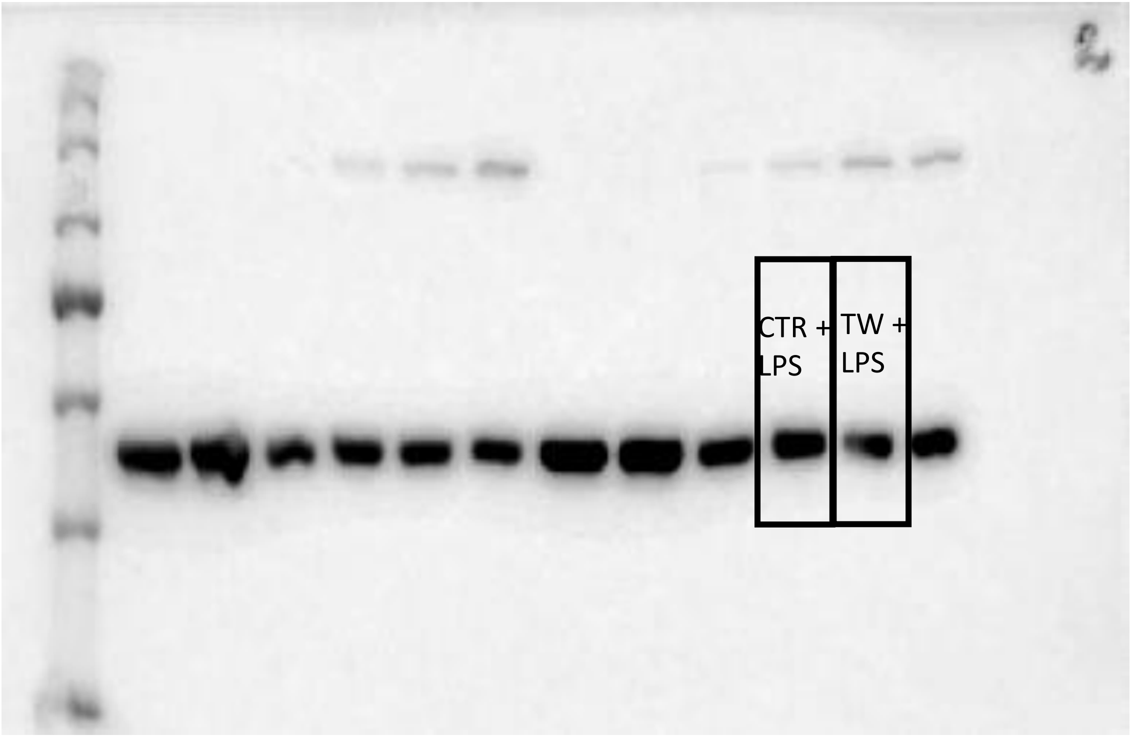

Supplement: Supplementary file 1 — Supplementary Information. [file 41598_2020_79394_MOESM1_ESM.pdf]
